# Catalytic generation of alkoxy radicals from unfunctionalized alcohols

**DOI:** 10.1039/d0sc04542j

**Published:** 2020-09-21

**Authors:** Elaine Tsui, Huaiju Wang, Robert R. Knowles

**Affiliations:** a Department of Chemistry , Princeton University , Princeton , NJ 08544 , USA . Email: rknowles@princeton.edu

## Abstract

Alkoxy radicals have long been recognized as powerful synthetic intermediates with well-established reactivity patterns. Due to the high bond dissociation free energy of aliphatic alcohol O–H bonds, these radicals are difficult to access through direct homolysis, and conventional methods have instead relied on activation of O-functionalized precursors. Over the past decade, however, numerous catalytic methods for the direct generation of alkoxy radicals from simple alcohol starting materials have emerged and created opportunities for the development of new transformations. This minireview discusses recent advances in catalytic alkoxy radical generation, with particular emphasis on progress toward the direct activation of unfunctionalized alcohols enabled by transition metal and photoredox catalysis.

## Abstract 2

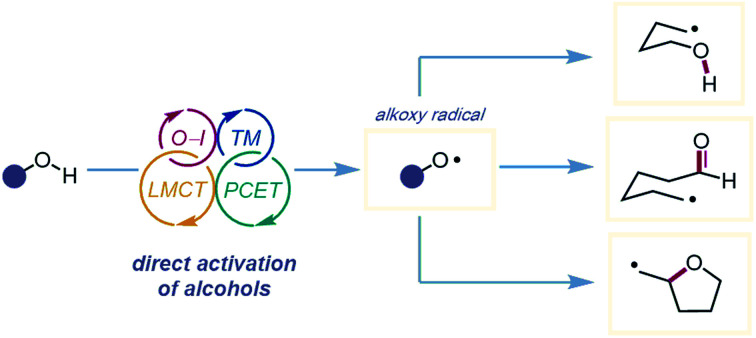
This review summarizes recent advances in the catalytic generation of alkoxy radicals from unfunctionalized alcohols and highlights current methods for O–H bond activation.

## Introduction

**Scheme 1:**
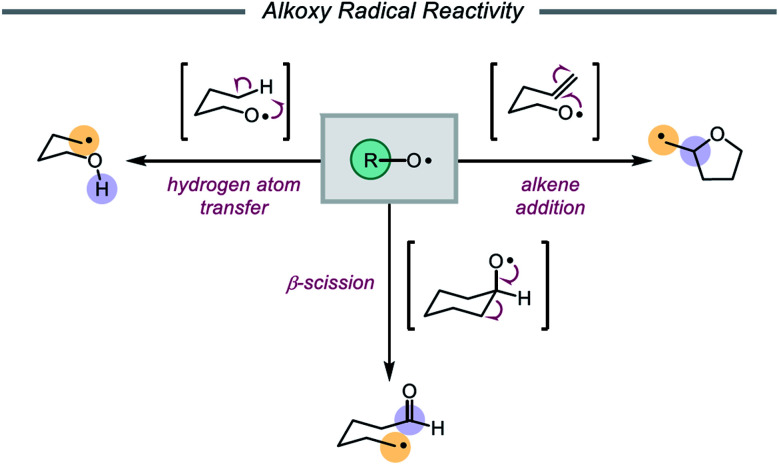
Elementary reactions of alkoxy radicals.

In 1911, Heinrich Wieland published one of the earliest reports that implicated the intermediacy of alkoxy radicals in an organic reaction, suggesting that alkoxy radicals were necessary in the formation of tetraphenyldiphenoxyethane from bis(triphenylmethyl)peroxide.^1^ Since then, alkoxy radicals have been recognized as versatile reactive intermediates and have been exploited in a wide variety of synthetic transformations.^2^ These electrophilic oxygen-centered radicals, consisting of an alkyl group bound to an oxygen radical center, lack the stabilization provided by mesomeric effects and spin density delocalization found in other O-centered radicals (*e.g.* aryloxy radicals). As a result, alkoxy radicals are a particularly high-energy species among heteroatom-centered radicals. Spectroscopic, mechanistic, and synthetic studies over the years have probed the reactivity of O-radicals,2c,^3^ finding that alkoxy radicals generally participate in one of three elementary reactions: (1) hydrogen atom transfer,^4^,^5^ (2) β-scission,^6^,^7^ and (3) alkene addition (Scheme 1).^8^,^9^

Of these three reaction classes, perhaps the most common application of alkoxy radicals is hydrogen atom transfer (HAT).^10^,^11^ Due to the propensity of O-centered radicals to form strong bonds to hydrogen atoms (O–H BDFE ≈ 105 kcal mol^–1^), hydrogen atom abstraction from comparatively weaker aliphatic C–H bonds (BDFE ≈ 98–102 kcal mol^–1^) is frequently observed.^12^,^13^ In particular, intramolecular 1,5-HAT allows for the direct abstraction of δ-C–H bonds and enables selective C–H functionalization, a synthetic strategy that has been exploited since the development of the Barton nitrite ester reaction^14^ and continues to be explored in modern catalytic methods.2e,2f Alkoxy radicals can also undergo homolytic cleavage of β-C–C bonds to furnish carbonyl products and C-centered radicals. As β-scission tends to favor cleavage of the C–C bond that will generate the most stabilized alkyl radical, the regioselectivity of such C–C cleavage events in unsymmetrical substrates is often both high and predictable.^15^–^17^ Lastly, alkoxy radicals engage in olefin addition reactions to form C–O bonds, most commonly in intramolecular cyclization reactions to afford cyclic ether scaffolds.^8^ While competition between these pathways does occur, careful design of substrates and control of reaction conditions can be utilized to bias one reaction type over another.^3^,^18^,^19^ Indeed, alkoxy radicals have long been successfully employed in the syntheses of complex molecules.^14^,^20^

**Scheme 2:**
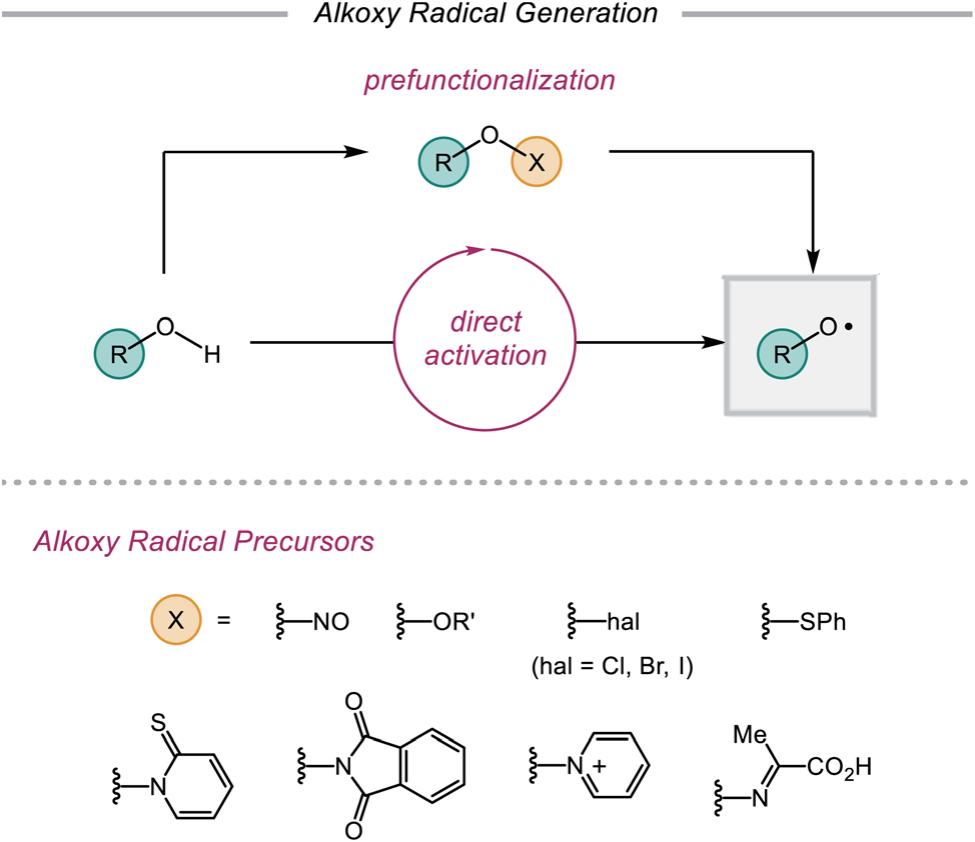
Methods for alkoxy radical generation.

Despite their synthetic potential, the generation of alkoxy radicals is nontrivial. Direct homolytic activation of alkanol O–H bonds has historically been challenging due to their high bond dissociation free energies and the frequent presence of weaker aliphatic C–H bonds in the same substrates. To circumvent these limitations, chemists have developed indirect strategies to access alkoxy radicals through the installation of weak oxygen-heteroatom bonds, such as those in nitrite esters,^14^,^21^ peroxides,15a,^22^ hypohalites,15c,^23^ sulfenates,^24^*N*-alkoxypyridine-2-thiones,^25^ and *N*-alkoxyphthalimides^26^ (Scheme 2). Prefunctionalization of native alcohols is not without drawbacks, from the inevitable loss of atom economy to the instability of many O-functionalized precursors. Chemo- and regioselective installation of such functionality in complex substrates bearing numerous reactive sites can also be challenging and generally necessitates the use of protecting groups. Moreover, these O–X bonds are homolyzed using thermolysis or UV photolysis, conditions that often limit functional group compatibility and the scope of subsequent transformations, preventing use of these surrogates in late-stage manipulations of complex molecules. While methods using milder radical initiators have been employed for O–X bond homolysis, these strategies often require efficient radical chain processes to function effectively.

Recently, modern catalytic methods have been developed to allow for alkoxy radical generation from bench-stable prefunctionalized substrates under milder conditions. For example, while classical strategies for activating the O–N bond of *N*-alkoxyphthalimide precursors require stoichiometric organotin reagents in the presence of a radical initiator,^26^ recently developed photocatalytic approaches have enabled the generation of alkoxy radicals from *N*-alkoxyphthalimides using a transient reductant that can be generated by low intensity irradiation with visible light.^27^,^28^ Analogous catalytic systems have also been reported that facilitate the reductive cleavage of *N*-alkoxypyridinium salts^29^–^32^ or the oxidative decomposition of oxyimino acids to unmask alkoxy radical intermediates.^33^

In spite of major advances in the installation and activation of alkoxy radical surrogates, prefunctionalization of native alcohols necessitates circuitous synthetic steps and generates stoichiometric byproducts. The direct catalytic activation of free alcohols, which bypasses prefunctionalization, has inherent advantages. The development of photoredox catalysis and the expansion of transition-metal catalysis over the last decade have led to the advent of new methodologies that have overcome limitations of classical alkoxy radical generation. Many aspects of alkoxy radicals have been reviewed over the years, with recent reviews examining different types of alkoxy radical-mediated transformations.2d–f The purpose of this review is to highlight recent advances in the development of catalytic methods for alkoxy radical generation from unfunctionalized alcohols, including (1) *in situ* generation of O–I species, (2) transition metal-mediated homolysis, (3) ligand-to-metal charge transfer (LMCT), and (4) proton-coupled electron transfer (PCET). Across these examples, we showcase powerful strategies for O–H bond activation, emphasizing how these mild methods for alkoxy radical generation have facilitated the development of myriad transformations mediated by alkoxy radicals, each with increasing complexity and functional-group compatibility. Ultimately, this review endeavors to summarize the advances in this area and to discuss current challenges and opportunities that lie ahead.

## *In situ* generation of O–I species

The use of organohypervalent iodine compounds for the generation of alkoxy radicals from alcohols has been investigated since the development of the Suárez system.2c,^34^ Comprising PhI(OAc)_2_ and I_2_, this reagent combination has been used as a strong oxidant, enabling *in situ* formation of a weak O–I bond that is generally photolyzed under visible-light irradiation to furnish an alkoxy radical intermediate. Various iodine(iii) compounds have been utilized to facilitate 1,5-HAT, β-scission, and alkene addition reactions, particularly in the context of steroidal and carbohydrate derivatives.^35^ However, in the same vein as classical reagents for O-radical generation, such as Pb(OAc)_4_, Ag_2_S_2_O_8_, HgO/I_2_, and (NH_4_)_2_[Ce(NO_3_)_6_], the PhI(OAc)_2_/I_2_ system has traditionally suffered from limited functional group compatibility, as the use of strong oxidants results in competitive oxidations that are deleterious to reaction efficiency. In recent years, interest in integrating iodane chemistry with photoredox catalysis has emerged, and new strategies have been developed to generate alkoxy radicals while circumventing the shortcomings of classical systems.

**Scheme 3:**
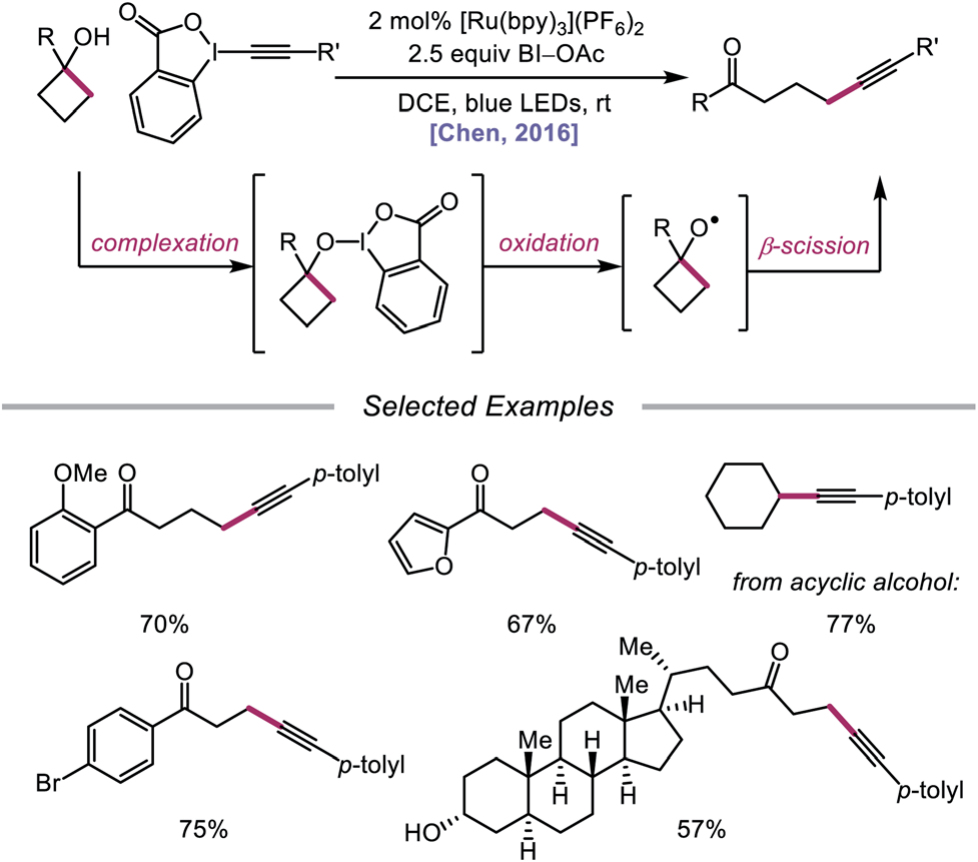
C–C bond cleavage and functionalization *via* CIR and photoredox catalysis.

**Scheme 4:**
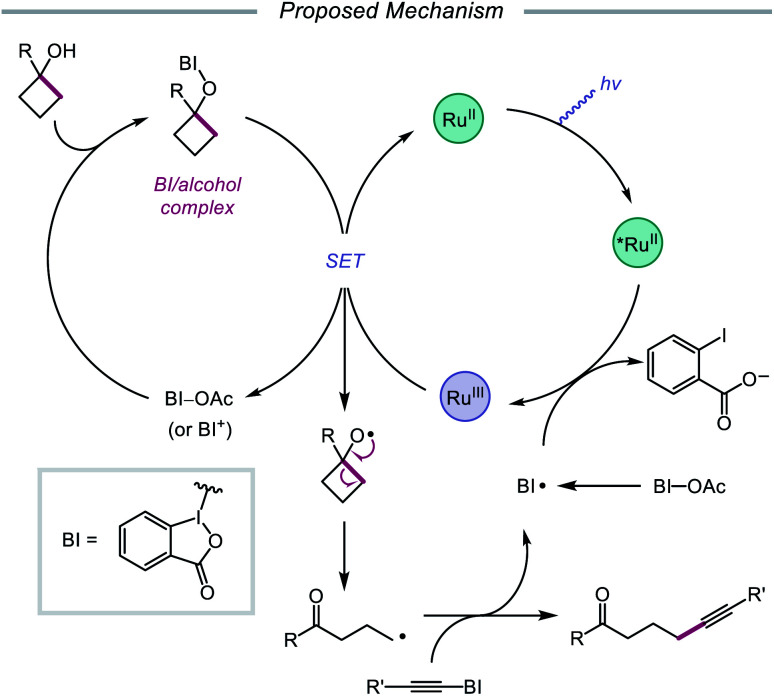
Proposed mechanism for ring-opening and alkynylation of cycloalkanols.

In 2016, Chen and coworkers disclosed a method for the activation of alcohols for C–C bond cleavage and subsequent alkynylation and alkenylation reactions.^36^ Central to this strategy is the use of cyclic iodine(iii) reagents (CIRs) under photoredox conditions to trigger formation of the requisite alkoxy radical, even though traditional non-cyclic iodine(iii) reagents, such as PhI(OAc)_2_, failed to give any desired product. A Ru(ii) photocatalyst and stoichiometric acetoylbenziodoxole (BI–OAc) are employed in the presence of a cycloalkanol and an alkynyl benziodoxole radical acceptor to furnish a variety of alkynyl ketones (Scheme 3). Cyclopropanols substituted with electron-rich or electron-deficient aryl groups are competent substrates, and electron-rich cyclobutanols are also smoothly converted to functionalized ketones. Steroidal cycloalkanols, including those with a free hydroxy group on the steroid core structure, are well tolerated under these reaction conditions, with ring-opening occurring only at the strained cycloalkanol moiety. Notably, linear alcohols also undergo β-scission and addition to not only alkynyl benziodoxoles but also vinyl carboxylates, which serve as precursors to alkenylated products. The authors propose that photoexcited Ru(ii) is initially oxidized to Ru(iii), a species that then oxidizes the O–I bond of an *in situ* formed benziodoxole/alcohol complex (Scheme 4). The alkoxy radical formed after oxidation undergoes C–C bond cleavage to give a distal alkyl radical that can add to a radical acceptor, releasing the alkynylation or alkenylation adduct and benziodoxole radical. The latter is expected to reoxidize excited-state Ru(ii) back to Ru(iii).

**Scheme 5:**
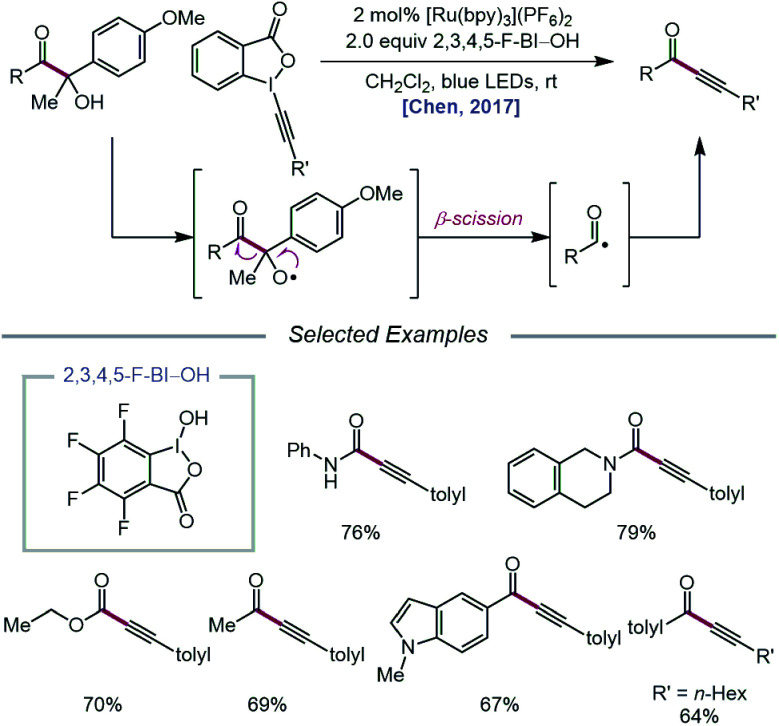
Carbonyl–C(sp^3^) bond cleavage and alkynylation for the synthesis of ynoates, ynamides, and ynones.

In 2017, the Chen group extended the use of CIRs to achieve carbonyl–C(sp^3^) bond cleavage and alkynylation of β-amide, β-ester, and β-ketone alcohols under photoredox conditions (Scheme 5).^37^ Designed around a similar mechanism to that proposed in their earlier work, this protocol for selective cleavage of carbonyl–C(sp^3^) bonds, which previously required UV light irradiation^38^ or transition-metal catalysts under high temperatures,^39^ utilizes β-carbonyl alcohols as substrates for alkoxy radical-mediated β-scission. Unlike their 2016 report, it was found that for many cases, BI–OAc does not give the optimal yield of ynamide, ynoate, or ynone products, but 2,3,4,5-F-BI–OH as the CIR results in higher yields with shorter reaction times. The authors postulate that electron-deficient CIRs have more positive reduction potentials relative to BI–OAc, which in turn enables more efficient quenching of photoexcited Ru(ii) to Ru(iii). Additionally, electron-deficient benziodoxoles are demonstrated to decrease the oxidation potential of the CIR/alcohol complex (*E*oxp = 0.93 V *vs.* SCE in MeCN for 3,4-F-BI–OAc/alcohol) relative to the BI/alcohol complex (*E*oxp = 1.06 V *vs.* SCE in MeCN) and the free β-carbonyl alcohol (*E*oxp = 1.69 V *vs.* SCE in MeCN), suggesting that the decreased oxidation potentials correlate with superior alcohol activation and alkoxy radical generating ability. The use of CIRs for O–H activation was similarly applied to P–C(sp^3^) bond cleavage in α-phosphorus alcohols to synthesize phosphonoalkynes.^40^

**Scheme 6:**
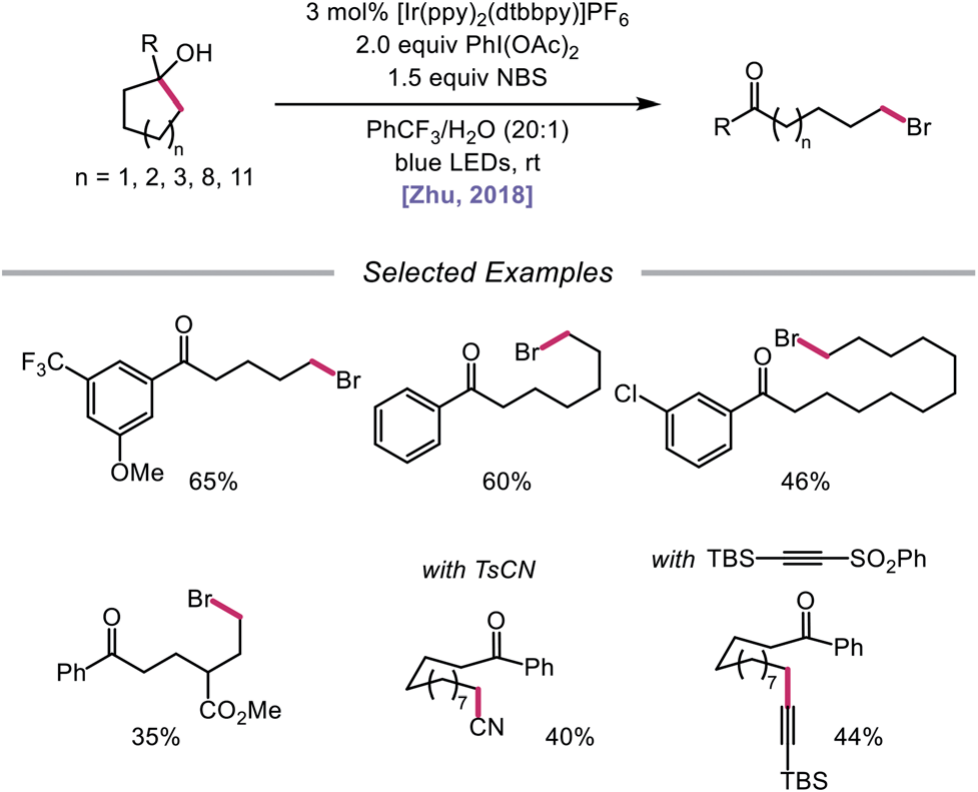
Visible light-promoted ring-opening and functionalization of cycloalkanols.

**Scheme 7:**
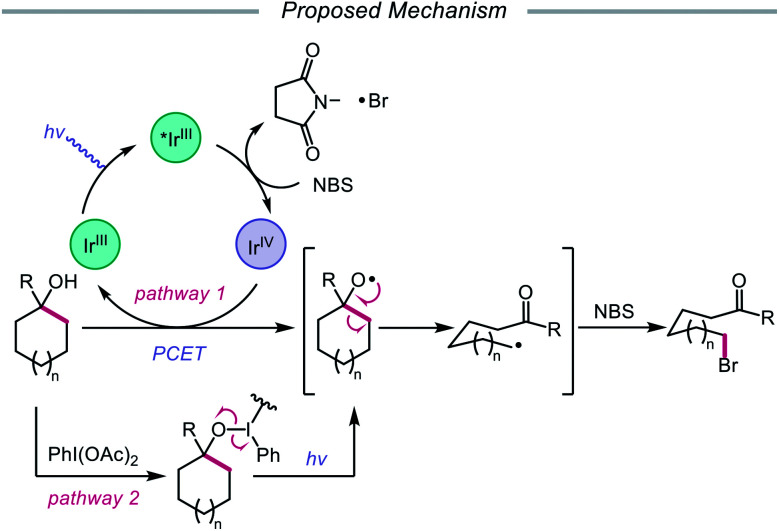
Postulated reaction pathways for ring-opening bromination using Ir(iii) photocatalyst and PhI(OAc)_2_.

**Scheme 8:**
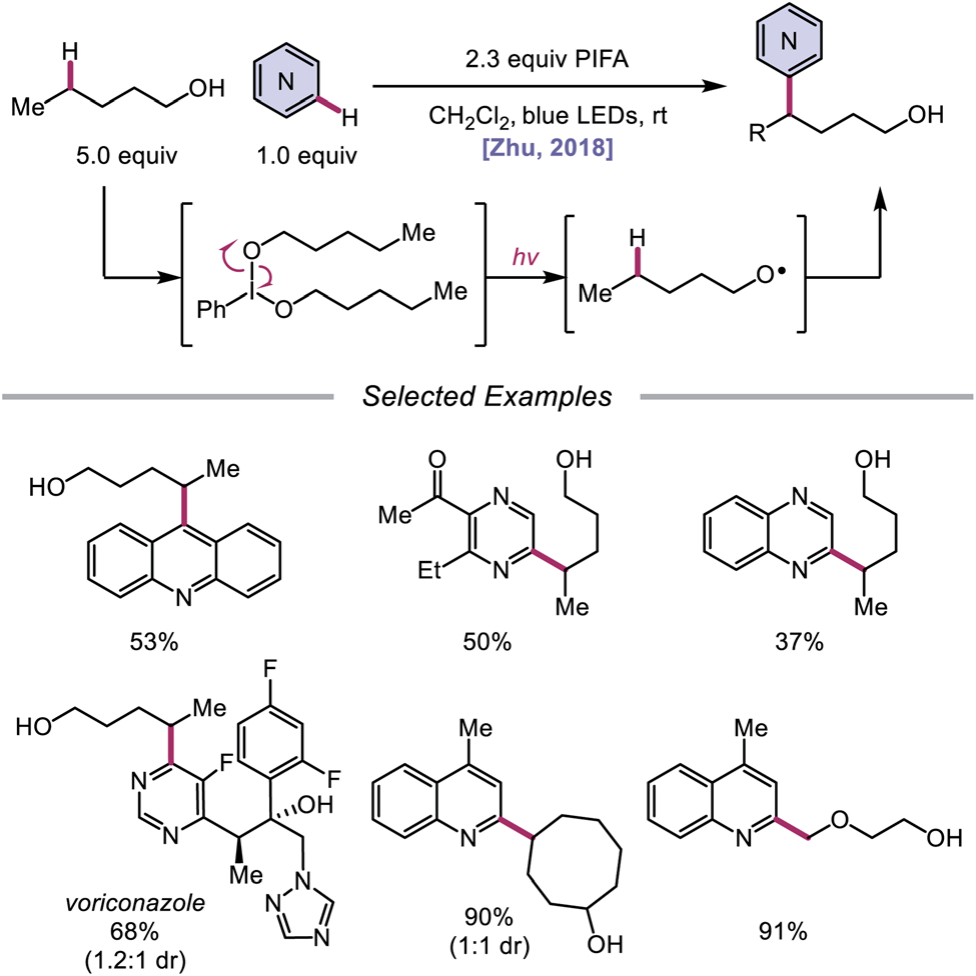
Metal-free heteroarylation of unactivated δ-C–H bonds through direct O–I bond homolysis.

In addition to oxidative cleavage of an alkoxyiodo intermediate, direct homolysis of an *in situ* formed O–I bond has been invoked in more recent mechanisms involving alkoxy radical formation. Zhu and coworkers demonstrated in 2018 that ring-opening bromination of unstrained cycloalkanols can be accomplished using an Ir(iii) photocatalyst, PhI(OAc)_2_, and *N*-bromosuccinimide (NBS) as the bromine source under visible light irradiation (Scheme 6).^41^ This protocol can be employed to construct distally brominated ketones from cyclopentanol, cyclohexanol, cycloheptanol, cyclododecanol, and cyclopentadecanol derivatives *via* alkoxy radical-mediated β-scission and bromination of the ring-opened C-centered radical. Additionally, substitution of NBS with tosyl cyanide and alkynyl sulfone allows for distal cyanation and alkynylation, respectively, through an analogous ring-opening mechanism. The authors propose that two pathways for O–H activation are operative (Scheme 7): (1) proton-coupled electron transfer involving Ir(iv) as an oxidant and succinimide anion as a weak base, and (2) formation of an alkoxyiodobenzene complex and visible light-induced O–I bond homolysis. Later that year, the Zhu group reported a related metal-free system for the alcohol-directed heteroarylation of C(sp^3^)–H bonds using phenyliodine bis(trifluoroacetate) (PIFA) as the only reagent (Scheme 8).^42^ While not catalytic, this strategy serves as an example of direct visible light-promoted O–I bond cleavage in the absence of a photoredox catalyst. It is postulated that a mixture of free alcohol and PIFA leads to formation of dialkoxyiodobenzene, which is confirmed by NMR experiments, and direct irradiation of this intermediate results in O–I bond homolysis to form the desired alkoxy radical. Intramolecular HAT affords the alkyl radical that can add into an *N*-heteroarene to furnish Minisci-type products, including functionalized acridine, pyrazine, quinoxaline, and drug molecules, such as voriconazole.

**Scheme 9:**
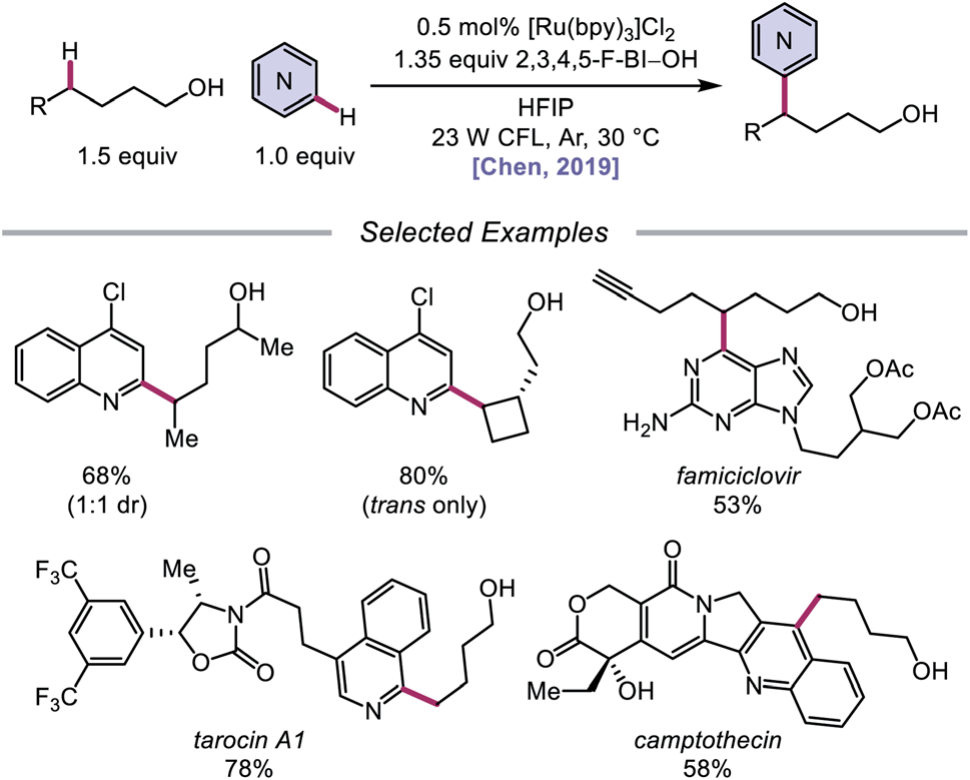
Photoredox-mediated remote heteroarylation of free alcohols.

**Scheme 10:**
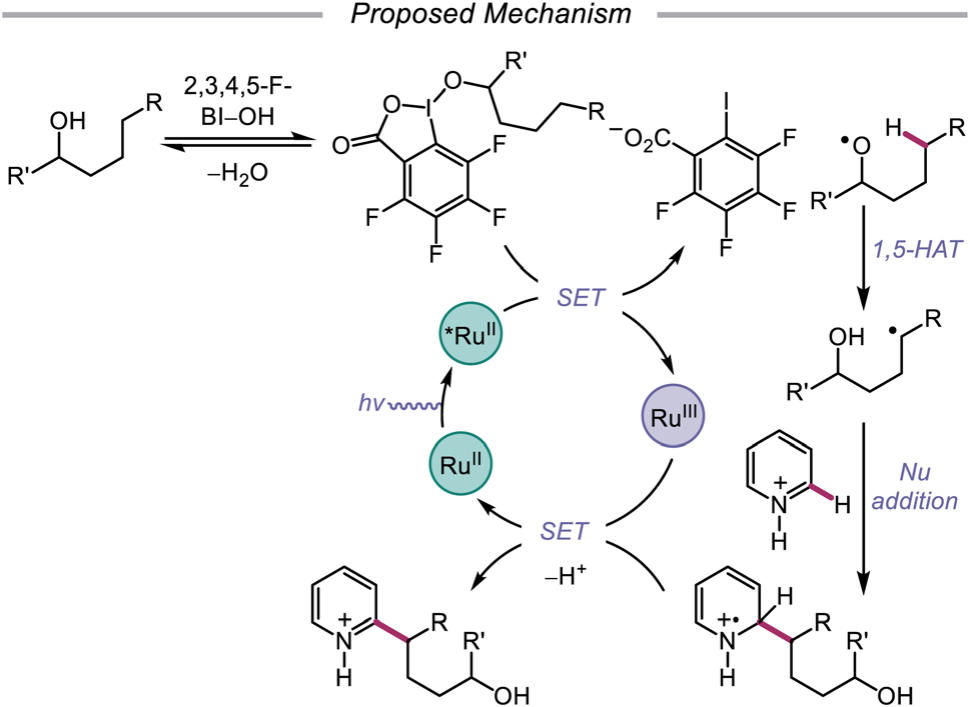
Proposed mechanism for the remote heteroarylation of free alcohols *via* photoredox catalysis.

Alternatively, in 2019, Chen and coworkers reported a photocatalytic strategy for the C(sp^3^)–H bond heteroarylation of alcohols using a Ru(ii) catalyst and 2,3,4,5-F-BI–OH (Scheme 9).^43^ This method enables efficient alkoxy radical formation and δ-C–H functionalization of primary and secondary alcohols, even when using just 1.5 equivalents of alcohol substrate (as opposed to the system reported by Zhu *et al.*), and has allowed for the alkylation of complex drug molecules, such as famciclovir, tarocin A1, and camptothecin. Mechanistic investigations suggest that 2,3,4,5-F-BI–OH can undergo alcoholysis with 1-butanol to give isolable 2,3,4,5-F-BI–OBu, an intermediate which gives the expected alkylated product under reaction conditions and has been shown to quench excited-state Ru(ii). It is, therefore, proposed that upon *in situ* formation, the new 2,3,4,5-F-BI–OR complex is activated *via* one-electron reduction by photoexcited Ru(ii) to give a benzoate anion and the alkoxy radical (Scheme 10). 1,5-HAT furnishes an alkyl radical, which reacts with *N*-heteroarenes through a Minisci-type reaction, and Ru(iii) oxidizes the resulting adduct to regenerate Ru(ii) and give the final product. This protocol for alkoxy radical generation was later extended to a related β-scission-promoted C–H bond alkylation of various *N*-heteroarenes.^44^

## Transition metal-mediated homolysis

Stoichiometric transition metal salts have been employed for single-electron oxidations of alcohols since the 1960s, usually apropos the oxidations of strained cycloalkanols, releasing ring strain through the formation of ring-opened products. For example, Schaafsma and coworkers reported that Cu(ii) and Fe(iii) salts can induce the oxidative ring-opening of cyclopropanone hemiacetal, forming β-propionate radical, which can then dimerize or react with a radical acceptor.^45^ Moreover, Roček found that Mn(iii), V(v), and Ce(iv) can serve as one-electron oxidizing agents for cyclobutanol,^46^ and in 1991, Narasaka disclosed the use of Mn(iii) tris(2-pyridinecarboxylate) for the generation of β-keto radicals through O–H activation of cyclopropanol derivatives, demonstrating their efficient addition to silyl enol ethers.^47^ More recently, focus has shifted from stoichiometric methods to the development of transition metal-catalyzed strategies for accessing alkoxy radicals. The key to a metal catalyst's success is its ability to turn over—namely, its capacity to be reoxidized to its active form as ligand exchange with the alcohol substrate occurs, necessitating the use of oxidants in the reaction. Early examples of such systems include AgNO_3_/Na_2_S_2_O_8_, used by Minisci and Surzur to activate acyclic alcohols for heteroarylation,^48^ and AgNO_3_/(NH_4_)_2_S_2_O_8_, employed by Narasaka for ring-opening and C(sp^3^)–C(sp^3^) bond functionalization of cyclopropanols.^49^ Since then, catalysts such as Ag(i), Mn(iii), and Cu(ii) have shown promise in the homolytic activation of alcohol O–H bonds and have been exploited in C–C bond cleavage, C–H bond functionalization, and C–O bond-forming reactions.

**Scheme 11:**
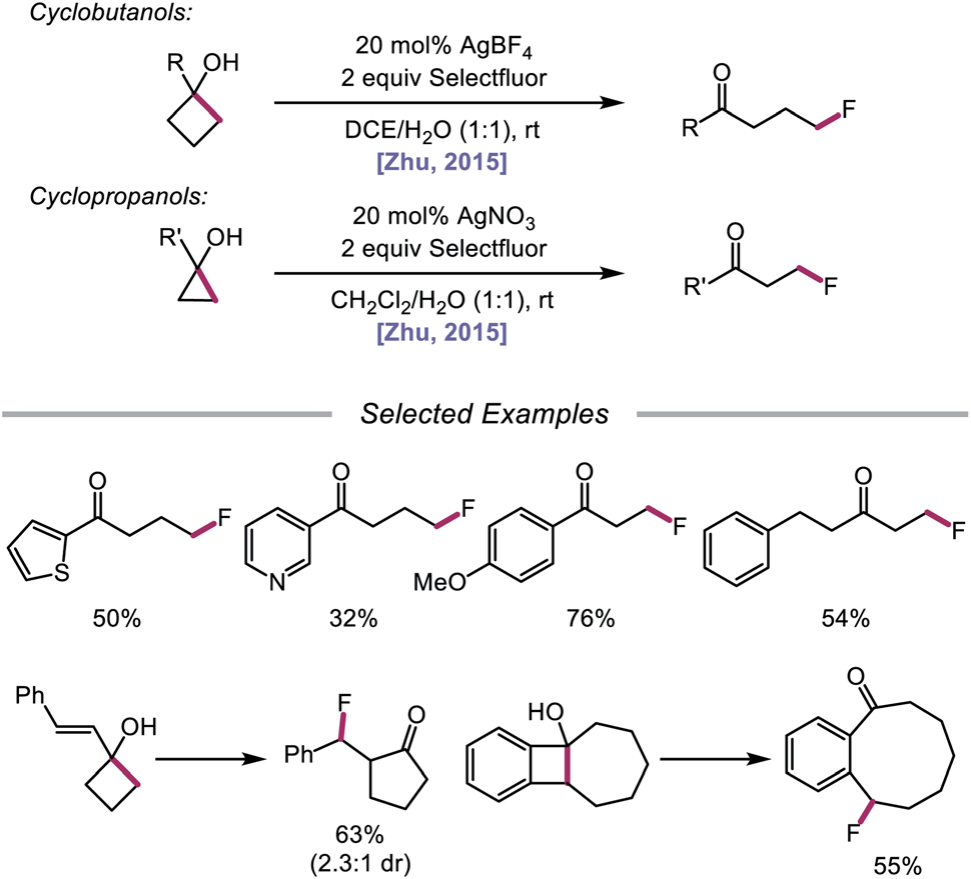
Silver-catalyzed ring-opening and fluorination of cyclopropanols and cyclobutanols.

**Scheme 12:**
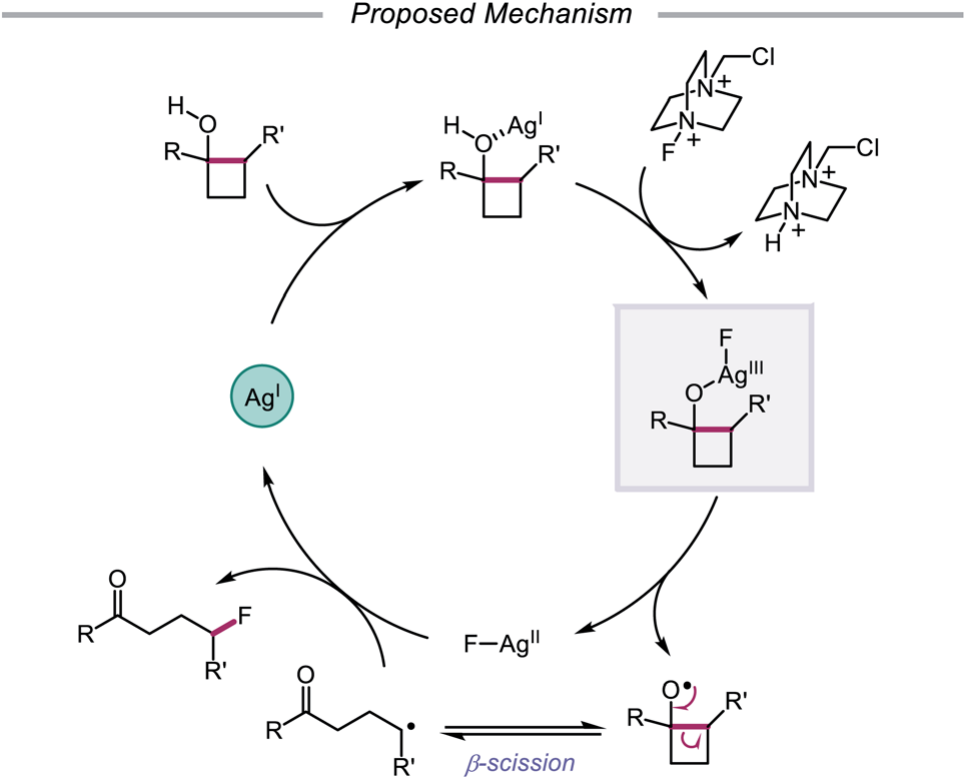
Proposed mechanism for silver-catalyzed ring-opening and fluorination.

In 2015, Zhu and coworkers reported a protocol for the ring-opening and fluorination of cyclopropanols and cyclobutanols that relies on a Ag(i) catalyst, Selectfluor as the fluorinating reagent and oxidant, and a biphasic solvent system (Scheme 11).^50^,^51^ These conditions can accommodate electron-rich and electron-deficient tertiary cyclobutanols, furnishing γ-fluorinated ketones, such as thienyl and pyridyl ketones. Additionally, this protocol has been adapted for the ring expansion of cyclobutanol to cyclopentanone and the expansion of a fused bicyclic framework to the corresponding nine-membered fluorinated ketone. Upon switching the optimal Ag(i) salt from AgBF_4_ to AgNO_3_, the authors report that facile ring-opening and fluorination of cyclopropanols can also be achieved. Mechanistic studies rule out base-assisted ring-opening *via* a polar mechanism as well as silver-assisted C–C bond cleavage through a Ag(i) insertion and reductive elimination pathway, suggesting the relevance of a silver-catalyzed radical mechanism. Accordingly, the authors propose that the alcohol substrate coordinates with the Ag(i) salt and reacts with Selectfluor to afford a Ag(iii)–F complex (Scheme 12). Homolysis of the Ag–O bond releases Ag(ii) and the alkoxy radical, which undergoes β-fragmentation to the distal alkyl radical. This C-centered radical intercepts Ag(ii)–F to form the fluoroketone product, regenerating Ag(i) in the process. At the time of this report, the groups of Loh and Murakami independently disclosed similar catalytic systems.^52^,^53^ Loh and coworkers additionally demonstrated that these conditions can be used for the ring-opening and fluorination of less strained cycloalkanols, including cyclopentanol and cyclohexanol, at the cost of reaction efficiency. Zhu and coworkers later extended silver-catalyzed ring-opening to chlorination reactions through the use of K_2_S_2_O_8_ as an oxidant and *N*-chlorosuccinimide as the chlorinating reagent; more recently, the Parsons group employed a similar AgNO_3_/K_2_S_2_O_8_ reaction system to achieve C–C bond cleavage and demethylation of azetidinols, forming α-amino ketone products as part of this alkoxy radical-mediated deconstructive protocol.^54^

**Scheme 13:**
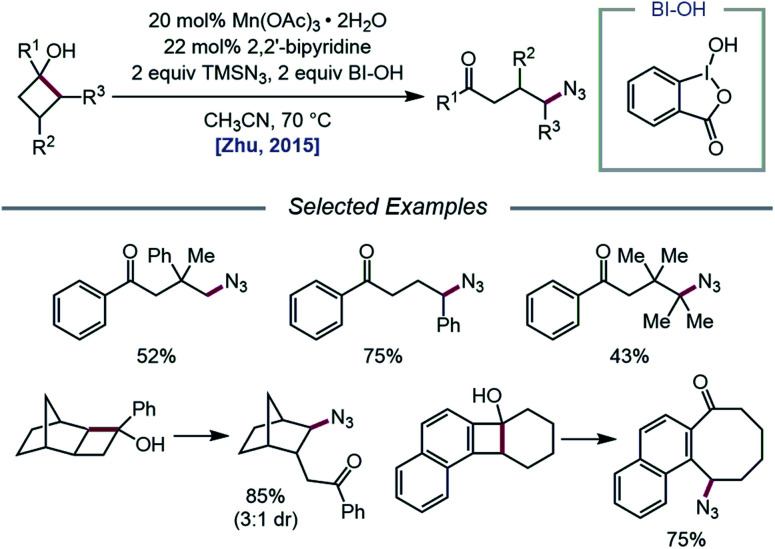
Manganese-catalyzed ring-opening and azidation of cyclobutanols.

**Scheme 14:**
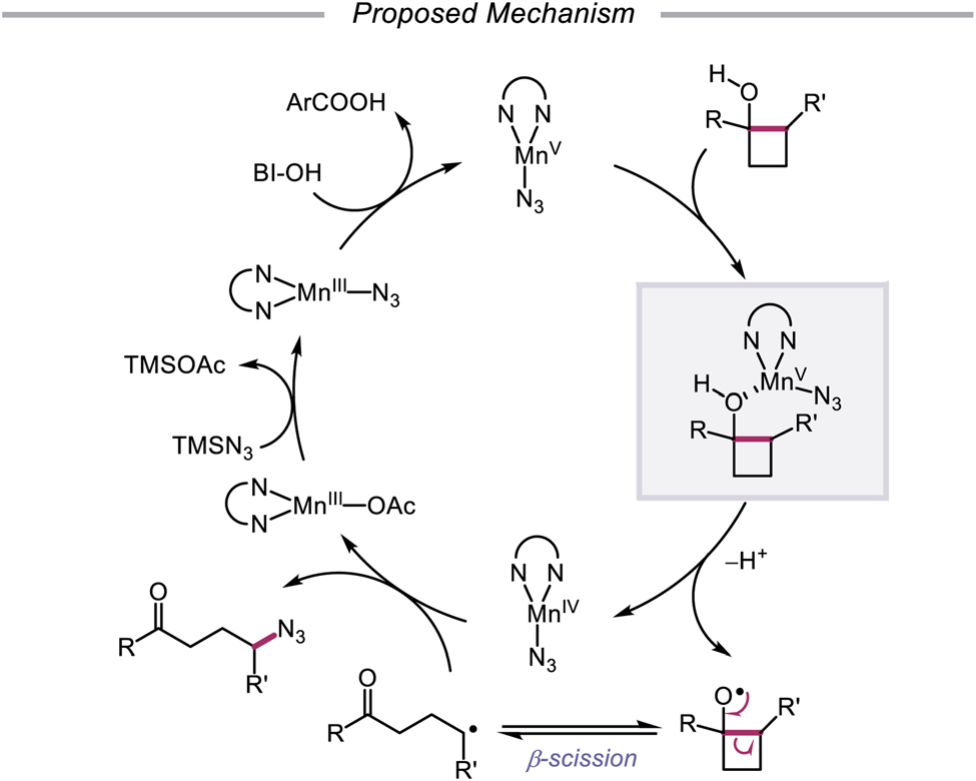
Prospective catalytic cycle for manganese-catalyzed ring-opening and azidation of cyclobutanols.

The Zhu group developed a manganese-catalyzed oxidative azidation of cyclobutanols later that year, enabling access to a diverse scope of linear and cyclic alkyl azides.^55^ During reaction optimization using 1-phenylcyclobutanol, it was found that all attempts at using a silver catalyst afforded undesired 1-tetralone, a product of intramolecular cyclization of the C-centered radical formed after ring-opening.^56^ Catalytic Mn(OAc)_3_, in the presence of BI–OH as the oxidant, 2,2′-bipyridine as a ligand, and TMSN_3_ as the azide source, was instead determined to give the optimal yield of ring-opened azide product (Scheme 13). Notably, the reaction can accommodate multisubstituted cyclobutyl rings as well as bicyclic scaffolds, which give rise to secondary or tertiary azides. In the absence of bipyridine ligand, medium-sized benzocyclic azides can also be formed by using a deconstructive ring-expansion strategy *via* cleavage of the C–C bond of the cyclobutanol moiety. The authors propose that after ligand exchange between Mn(OAc)_3_, bipyridine, and TMSN_3_, BI–OH oxidizes the metal complex to a high-valent Mn(v)–N_3_ species (Scheme 14). Complexation with cyclobutanol, followed by single-electron transfer, yields Mn(iv)–N_3_ and the cyclobutyloxy radical, which then undergoes β-scission to form a ring-opened alkyl radical. The nascent C-centered radical is intercepted by Mn(iv)–N_3_ to form the alkyl azide product, and further reactions with another molecule of TMSN_3_ and BI–OH turn over the catalytic cycle. Analogous catalyst systems have been developed for cyanation and alkynylation,^57^ chlorination,^58^ hydrazination,^59^ thiolation,^60^ and selenation.^61^

**Scheme 15:**
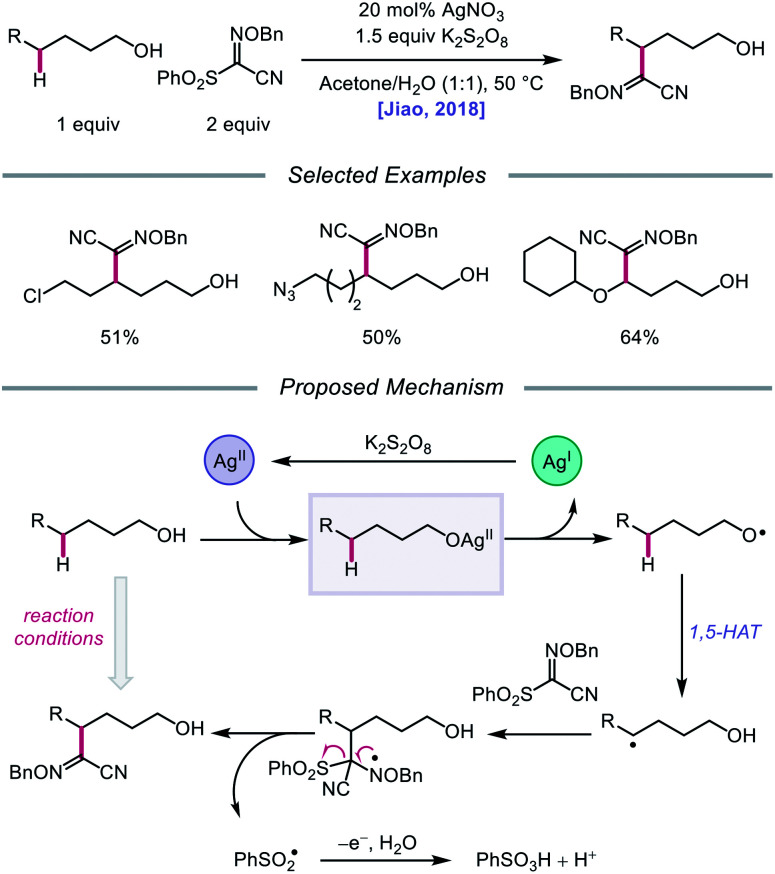
Silver-catalyzed functionalization of remote C(sp^3^)–H bonds of aliphatic alcohols.

Silver catalysis was again leveraged for alkoxy radical formation in a report by the Jiao group in 2018, this time to promote 1,5-HAT and subsequent C(sp^3^)–H bond functionalization to form oxime ether products.^62^ The authors identify AgNO_3_ and K_2_S_2_O_8_ as the optimal metal/oxidant pair to use for selective δ-C–H abstraction and a sulfonyl oxime ether reagent as the radical acceptor for the transformation (Scheme 15). The reaction tolerates primary and secondary alcohols, including substrates bearing halogen and azide substituents. Additionally, the presence of an oxygen atom at the *ε* position of the alkanol is critical to obtaining higher yields, presumably due to added stability and nucleophilicity conferred to the adjacent C-centered radical. When a β-substituted alkanol was judiciously subjected to the reaction conditions, a product resulting from β-scission and addition to the oxime ether acceptor was observed, consistent with an alkoxy radical-mediated pathway. As a result, during the course of the reaction, AgNO_3_ is postulated to be oxidized by K_2_S_2_O_8_ to Ag(ii), which coordinates with the alcohol substrate, and the resulting Ag(ii) complex undergoes homolysis to give the alkoxy radical and Ag(i) (Scheme 15). The O-centered radical participates in 1,5-HAT, furnishing an alkyl radical that adds to the acceptor, which induces fragmentation and release of a sulfonyl radical and the functionalized product. Jiao and coworkers recently applied similar alcohol activation conditions to an aqueous catalyst system for the regioselective O–H homolysis of 1,3-diols in the presence of DMSO as a hydrogen-bond acceptor, enabling distal C–C cleavage and 1,4-aryl migration.^63^

**Scheme 16:**
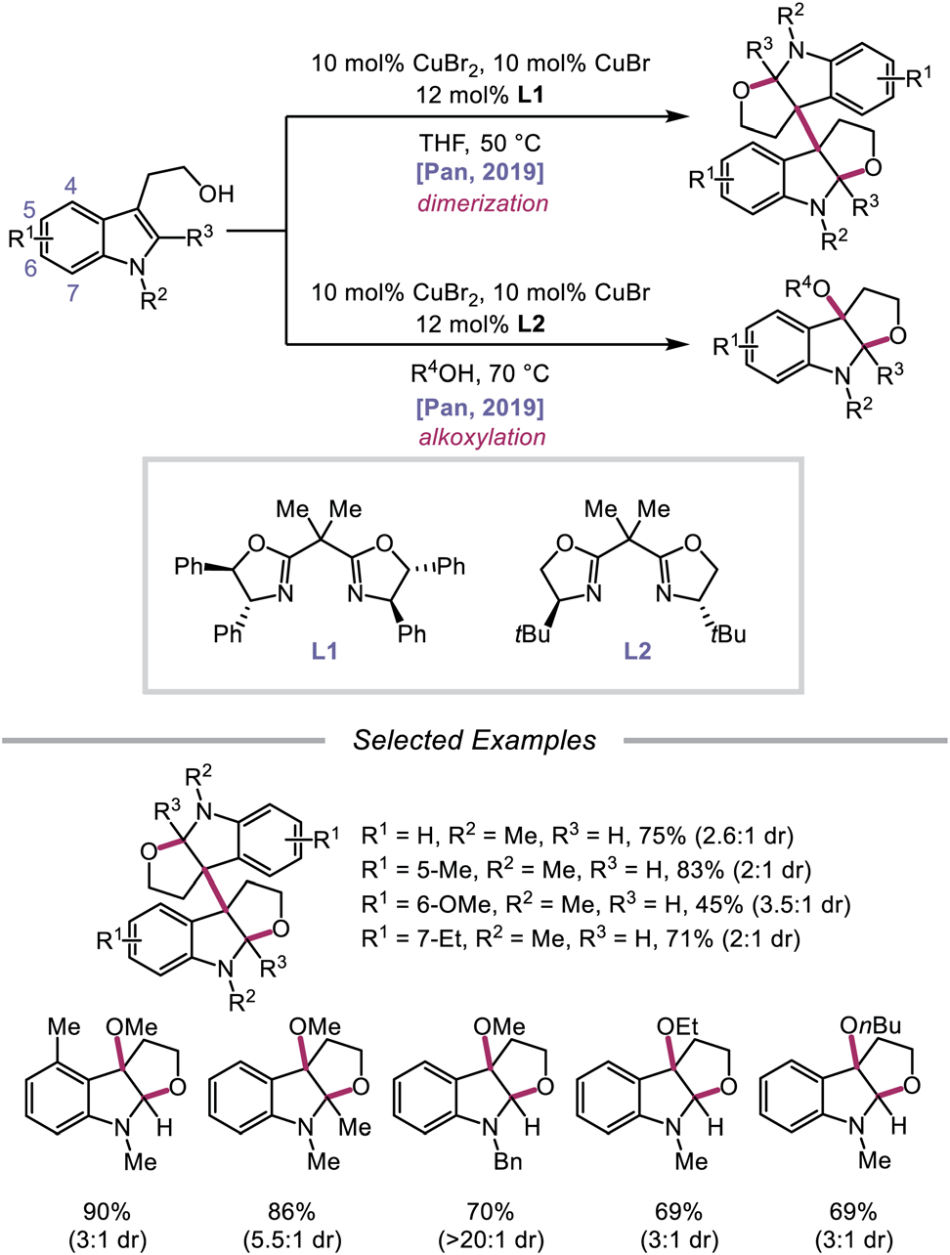
Copper-catalyzed alkoxy radical generation for the synthesis of furoindoline derivatives.

**Scheme 17:**
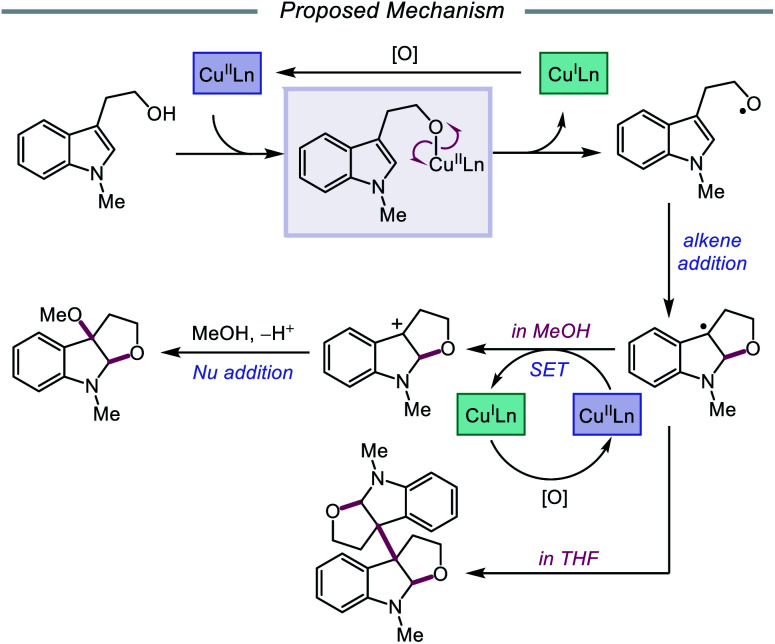
Postulated mechanism for copper-catalyzed alkoxy radical generation and alkene addition.

In 2019, Pan and coworkers disclosed a protocol encompassing a copper and oxazoline catalyst system for alkoxy radical generation and C–O bond formation to access 3a,3a′-bisfuro[2,3-*b*]indoline scaffolds and 3-alkoxy furoindolines from tryptophol derivatives (Scheme 16).^64^ Remarkably, this method does not require exogenous base, initiator, or oxidant, but the use of a dual Cu(i)/Cu(ii) catalyst combination and a bisoxazoline ligand has proven to be essential to reaction efficiency. When run under THF, the protocol affords good yields and moderate diastereoselectivities of C-radical dimerization products, tolerating electron-rich substituents at the 5-, 6-, and 7-positions of the indole ring. In contrast, when the reaction is run in a dilute protic alcohol solvent, such as methanol, the solvent can serve as a trapping reagent to generate a variety of substituted 3-alkoxy furoindolines. Results from mechanistic studies suggest that ligand exchange occurs between the alcohol substrate and the Cu(ii) complex to generate a Cu(ii)-alkoxide,^65^ followed by homolysis to form a Cu(i) species and an alkoxy radical (Scheme 17). Intramolecular alkene addition forms the cyclized intermediate containing an adjacent C-centered radical, which undergoes radical–radical dimerization in THF. Alternatively, the alkyl radical can be further oxidized by Cu(ii) to generate a carbocation, trapping the protic alcohol solvent to deliver the 3-functionalized furoindoline. While the role of CuBr in accelerating the reaction remains unclear, Cu(i) generated during the course of the reaction is proposed to be reoxidized to Cu(ii) by O_2_.

## Ligand-to-metal charge transfer

**Scheme 18:**
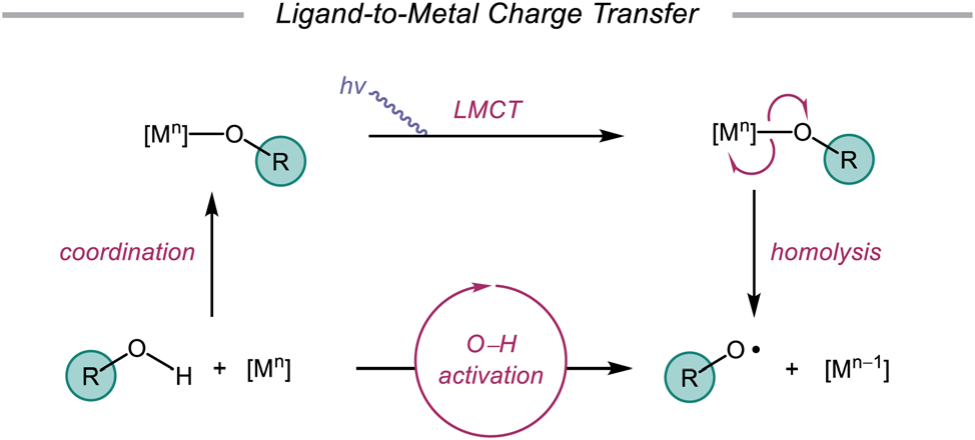
LMCT as a platform for alkoxy radical generation from unfunctionalized alcohols.

Advances made in visible-light photoredox catalysis over the past decade have opened new avenues for the generation and utilization of open-shell intermediates under mild reaction conditions.^66^ Many of the new technologies developed in this area rely on accessing the triplet excited state of a late transition-metal polypyridyl photocatalyst *via* photoinduced metal-to-ligand charge transfer (MLCT). An MLCT transition results in donation of an electron from a metal-centered orbital to a ligand-centered anti-bonding orbital. This excited-state complex, which can serve as both an oxidant or reductant, typically engages in single-electron transfer (SET) with the organic substrate, generating a radical intermediate that can be exploited in subsequent transformations. An alternative photoexcitation manifold, ligand-to-metal charge transfer (LMCT) involves complexes of transition metals with an empty valence shell, wherein an electron from a high-energy ligand orbital is excited to a low-lying metal-centered orbital.^67^ From the LMCT state, the metal–ligand bond is primed to homolyze, resulting in a formal reduction of the metal center and generation of a ligand-based radical. LMCT catalysis proceeds through sequential substrate coordination to the metal-based catalyst, LMCT excitation, and homolysis of the metal–ligand bond (Scheme 18). This method represents a strategy for substrate activation distinct from processes involving outer-sphere electron transfer events from an excited-state photocatalyst. In the case of O–H activation, LMCT promotes direct oxidation *via* the excitation event itself, providing a pathway for targeted oxidation of the transiently coordinated metal-alkoxide while leaving other functionality intact. Alkoxy radicals can, therefore, be selectively generated from simple alcohols. The Zuo group has found LMCT to be a powerful platform for alkoxy radical generation and has demonstrated the application of this strategy in a number of reports, a survey of which will be presented in this section of the review.

**Scheme 19:**
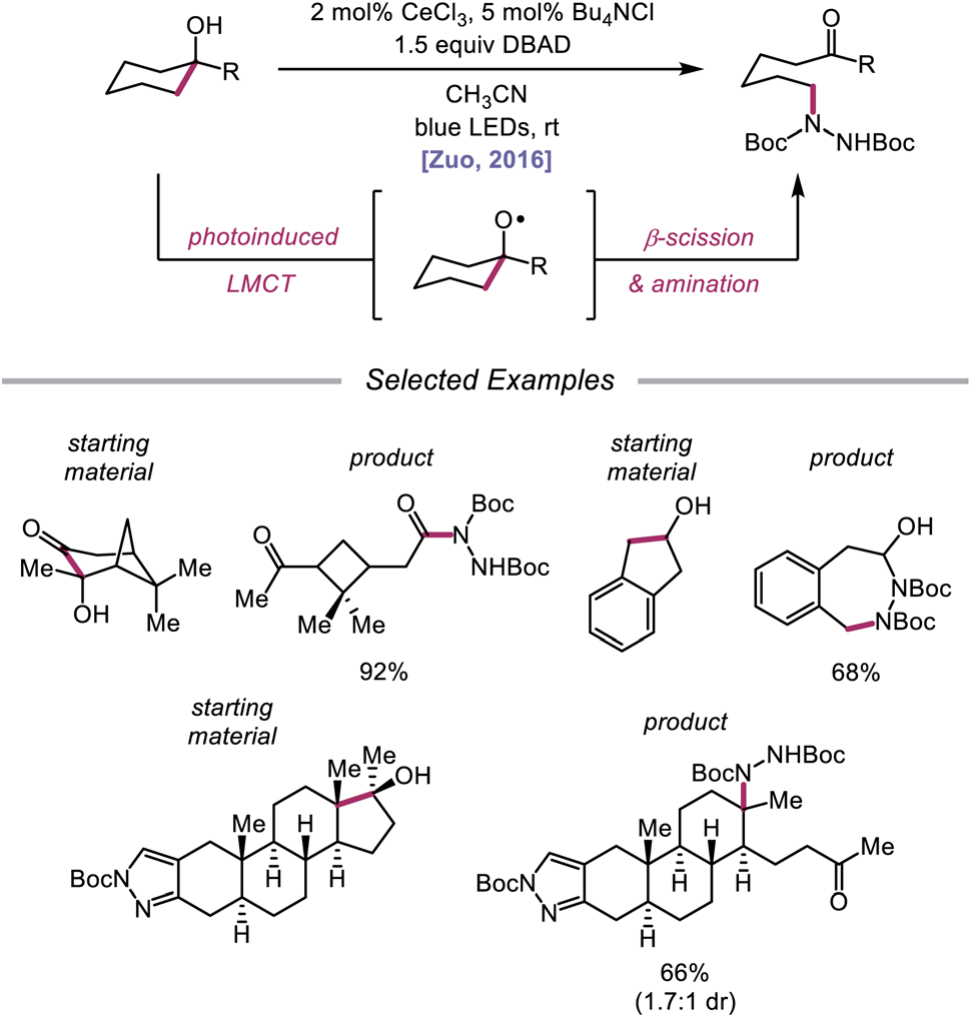
C–C bond cleavage and amination of cycloalkanols *via* cerium catalysis. DBAD, di-*tert*-butyl azodicarboxylate.

**Scheme 20:**
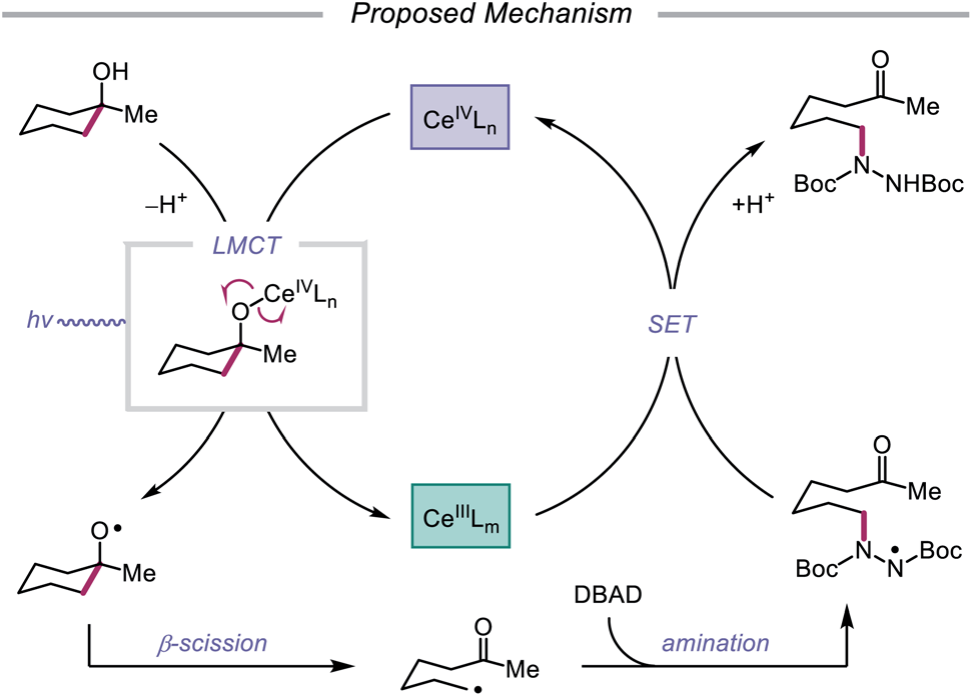
Proposed mechanism for C–C bond cleavage and amination of cycloalkanols through LMCT activation of O–H bonds.

Drawing inspiration from seminal work by Anna, Schelter, and coworkers,^68^ the Zuo group reported in 2016 the realization of photoinduced LMCT for O–H activation using a cerium-based catalyst system.^69^ In the presence of CeCl_3_ precatalyst and Bu_4_NCl under visible-light irradiation, cyclic alcohols undergo C–C bond β-scission and amination with azodicarboxylates to form distally aminated ketones (Scheme 19). Cycloalkanols of varying ring sizes are well tolerated in the reaction, including steroid- and terpenoid-derived cycloalkanols containing polar functionality. Remarkably, secondary alcohols, such as 2-indanol, are aminated using this protocol to furnish functionalized aldehydes, and this strategy can be employed for the synthesis of molecules with pharmaceutically relevant diazepine scaffolds. The authors propose the following mechanism2*d* (Scheme 20): Ce(iii) is initially oxidized to Ce(iv) by photoexcited azodicarboxylate, followed by *in situ* formation of a Ce(iv)–OR complex from the alcohol starting material.^70^ This Ce(iv)–alkoxide species then undergoes LMCT photoexcitation and subsequent homolysis to generate Ce(iii) and an alkoxy radical intermediate. C–C bond cleavage and ring-opening provide a carbonyl and distal C-centered radical, the latter of which couples with azodicarboxylate to form a nascent N-centered radical. It is postulated that this N-centered radical is reduced by Ce(iii) to afford the product and to turn over the catalytic cycle. Recently, the Zuo lab merged LMCT-mediated alkoxy radical generation with Lewis acid catalysis to achieve a one-step protocol for the C–C bond cleavage of ketones.^71^

**Scheme 21:**
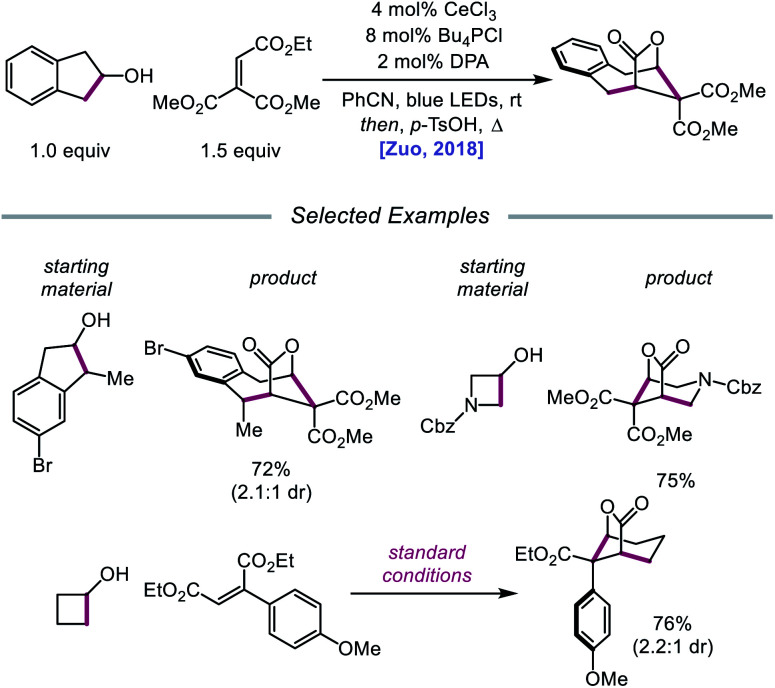
Cerium-catalyzed β-scission and ring-expansion of cycloalkanols for the formation of bridged lactones.

**Scheme 22:**
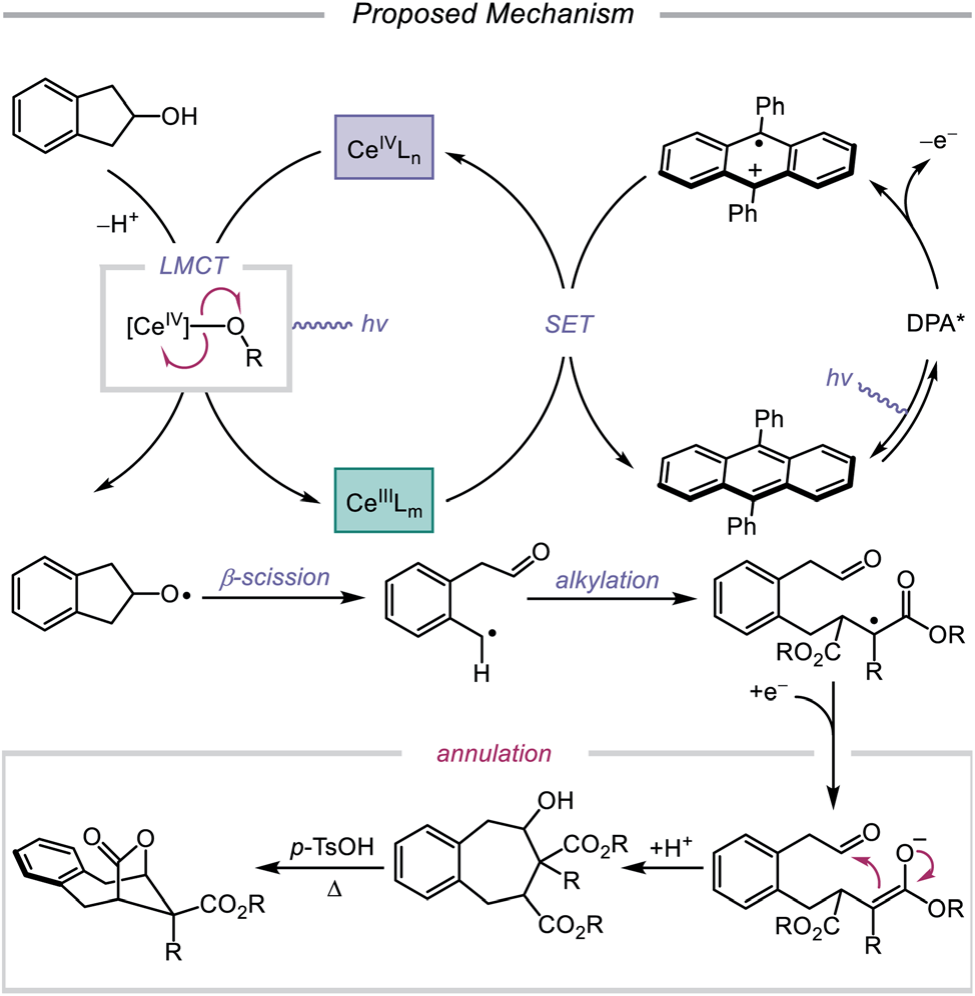
Prospective mechanism for the ring-expansion of cycloalkanols *via* dual photoexcitation.

In 2018, Zuo and coworkers extended this method of alkoxy radical generation to the synthesis of bridged lactones by leveraging cerium-catalyzed β-scission and ring expansion of cycloalkanols.^72^ Given the redox potential of their Ce(iii) catalyst (*E*_1/2_ (Ce^IV^/Ce^III^) = 0.40 V *vs.* SCE in MeCN), regeneration of photoactive Ce(iv) to turn over the catalytic cycle requires a sufficiently oxidizing radical intermediate, such as the N-centered radical employed in the authors' earlier alkoxy radical-mediated amination work. As a result, any catalytic system relying on a single cerium catalyst is constrained to a narrow selection of electron acceptors and transformations and cannot accommodate the single-electron reduction of common radical intermediates such as α-acyl radicals (*E*_1/2_ = –0.60 V *vs.* SCE in MeCN). To overcome this limitation, an electron shuttle with enough oxidizing power to oxidize Ce(iii) to Ce(iv) and enough reducing potential to quench the radical intermediate was investigated. The authors concluded that 9,10-diphenylanthracene (DPA) can serve as an efficient electron transfer catalyst and, together with the cerium photocatalyst, can enable the ring expansion of cycloalkanols with electron-deficient alkenes to form bridged lactones (Scheme 21). Results from UV-Vis absorption spectroscopy and Stern–Volmer quenching studies suggest that excited-state DPA plays a catalytic role in the reaction, rendering the reaction a two-photon process. More specifically, it is postulated that irradiation first triggers LMCT, promoting Ce–OR bond homolysis to form the alkoxy radical intermediate (Scheme 22). Cleavage of the α-hydroxy C–C bond furnishes an alkyl radical that then adds into an electron-deficient alkene, forming an α-acyl radical. Reduction of this nascent radical is achieved using photoexcited DPA (*E_1/2_ = –1.77 V *vs.* SCE in MeCN) to give an enolate and DPA˙^+^. An intramolecular aldol reaction facilitates annulation to a ring-expanded intermediate, which is then converted to a bridged lactone upon treatment with *p*-TsOH, while DPA˙^+^ (*E*_1/2_ = 1.13 V *vs.* SCE in MeCN) oxidizes Ce(iii) back to catalytically active Ce(iv). Substituted indanols, including those containing functional groups prone to transition-metal-catalyzed insertions, are amenable to the reaction, forming [4.2.1]-bridged lactones, while cyclobutanol derivatives are easily converted to a diverse range of [3.2.1] ring systems.

**Scheme 23:**
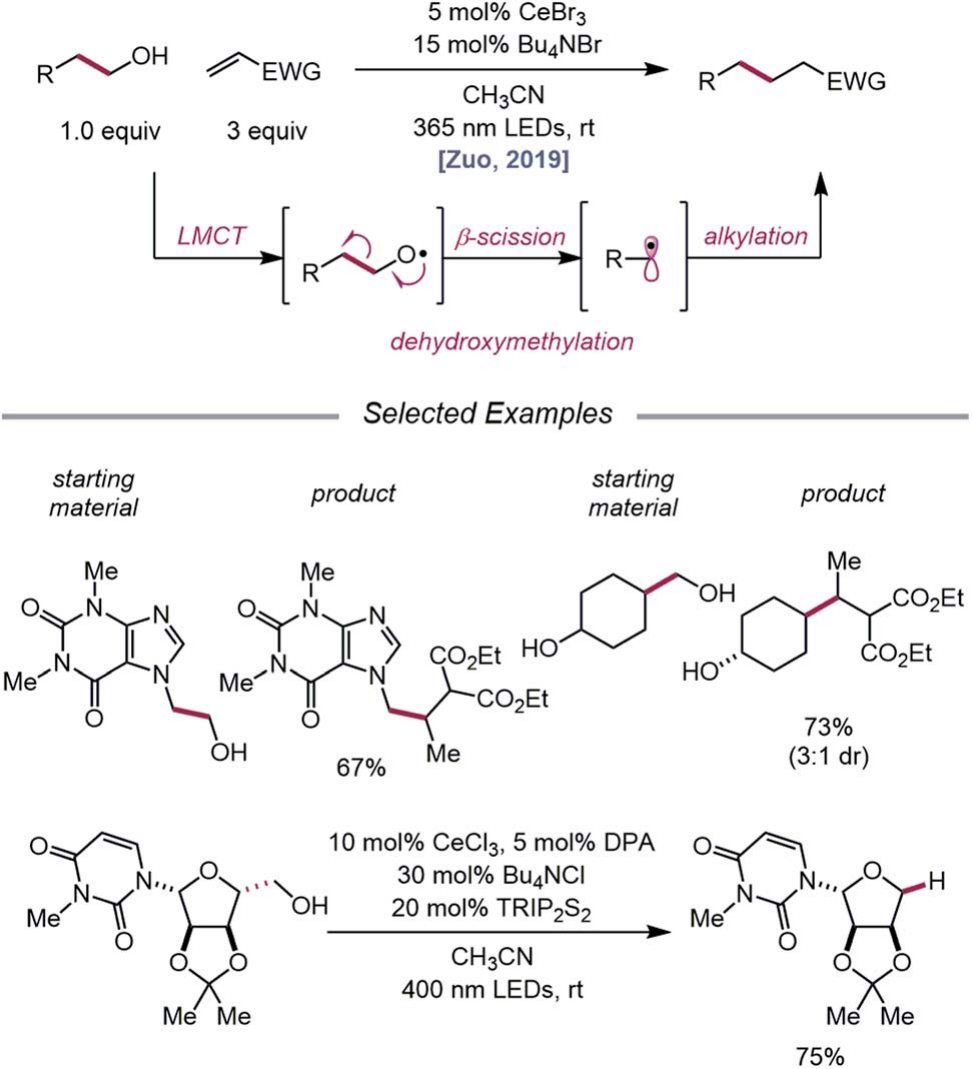
Cerium-catalyzed dehydroxymethylation of primary alcohols enabled by LMCT. TRIP_2_S_2_, 1,2-bis(2,4,6-triisopropylphenyl)disulfane.

The Zuo lab later disclosed an application of cerium-catalyzed β-scission to the dehydroxymethylation of alcohols and functionalization of these nucleophilic alkyl synthons in radical-mediated conjugate additions (Scheme 23).^73^ Through a double-excitation mechanism, C–C bond β-cleavage can be exploited to form alkyl radicals with concomitant extrusion of formaldehyde. A catalyst system comprising CeBr_3_ with Bu_4_NBr under 365 nm irradiation was found to be integral to the efficiency of the reaction, allowing for excitation of both the *in situ* formed Ce(iv)–alkoxide species to the LMCT excited state and the Ce(iii)–alkoxide complex. The excited-state of the Ce(iii)–alkoxide serves as a strong reductant and is capable of reducing the α-acyl radical to the desired product following alkyl radical addition to an electron-deficient alkene. Moreover, the authors note that for dehydroxymethylation to occur with primary alcohols, the corresponding alkoxy radicals would have to undergo β-scission faster than competitive 1,5-HAT. Interestingly, this cerium-based catalyst system generally favors dehydroxymethylation, as demonstrated in selectivity studies, and it is suggested that the weak interaction between alkoxy radicals and surrounding Lewis acidic cerium salts can promote the desired β-scission pathway. A variety of primary alcohols embedded in complex substrates, including protected uridine and adenosine, are amenable to the reaction; surprisingly, primary hydroxy groups are selectively dehydroxymethylated in the presence of secondary and tertiary alcohols in simple carbocycles. Beyond C(sp^3^)–C(sp^3^) bond formation, this protocol further leverages the generated alkyl radicals for hydrogenation, amination, alkenylation, and oxidation reactions.

**Scheme 24:**
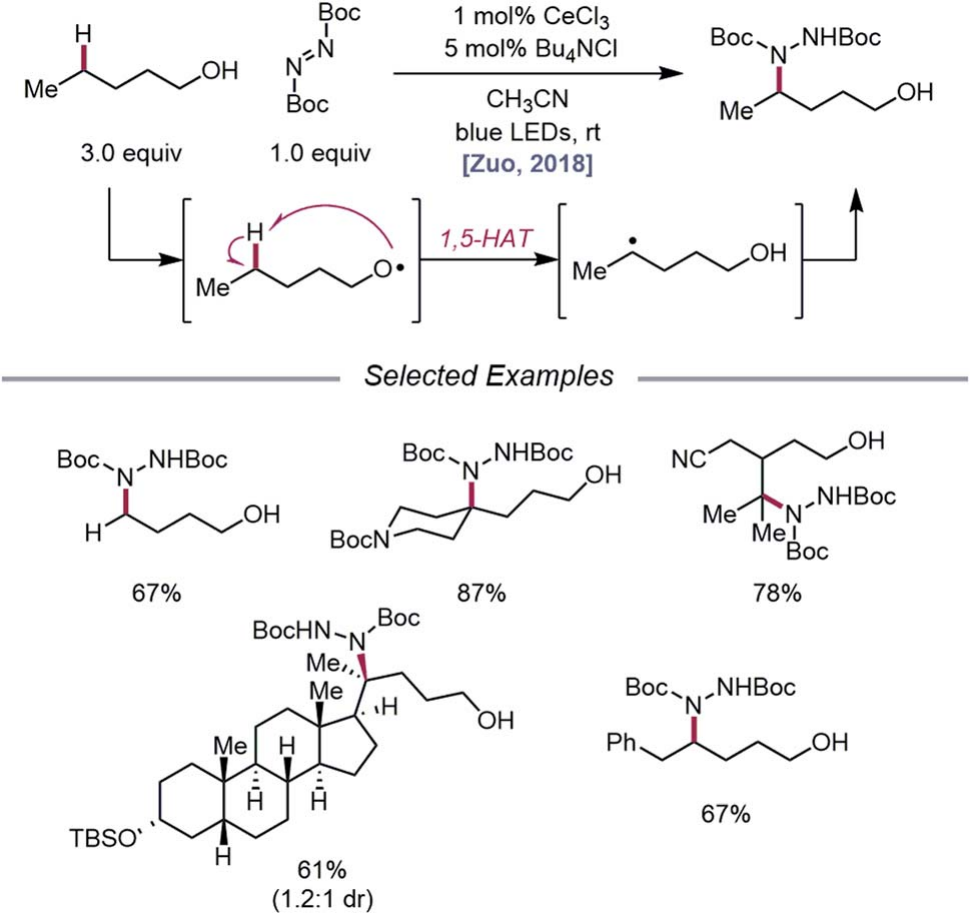
Alkoxy radical-mediated δ-C–H functionalization of alkanols *via* LMCT.

As LMCT was emerging as a method for realizing β-scission reactions, the Zuo group demonstrated in 2018 the advantages LMCT can provide for achieving remote C–H bond functionalization.^74^ This simple protocol employs catalytic CeCl_3_ and Bu_4_NCl to promote alkoxy radical formation *via* a photoexcited Ce(iv)–alkoxide species. Instead of undergoing β-scission, the resulting primary alkoxy radical engages in facile 1,5-HAT to form a δ-C-centered radical, which can couple with di-*tert*-butyl azodicarboxylate (DBAD) to construct a new C–N bond and deliver the desired amination product (Scheme 24). A variety of free alcohols, from 4-butanol to lithocholanyl alcohol, are functionalized in this manner. Notably, β-fragmentation is not a competitive pathway for the alcohol substrates studied, likely because these substrates lack substituents at the α- and β-carbons and are not generally prone to β-scission.

**Scheme 25:**
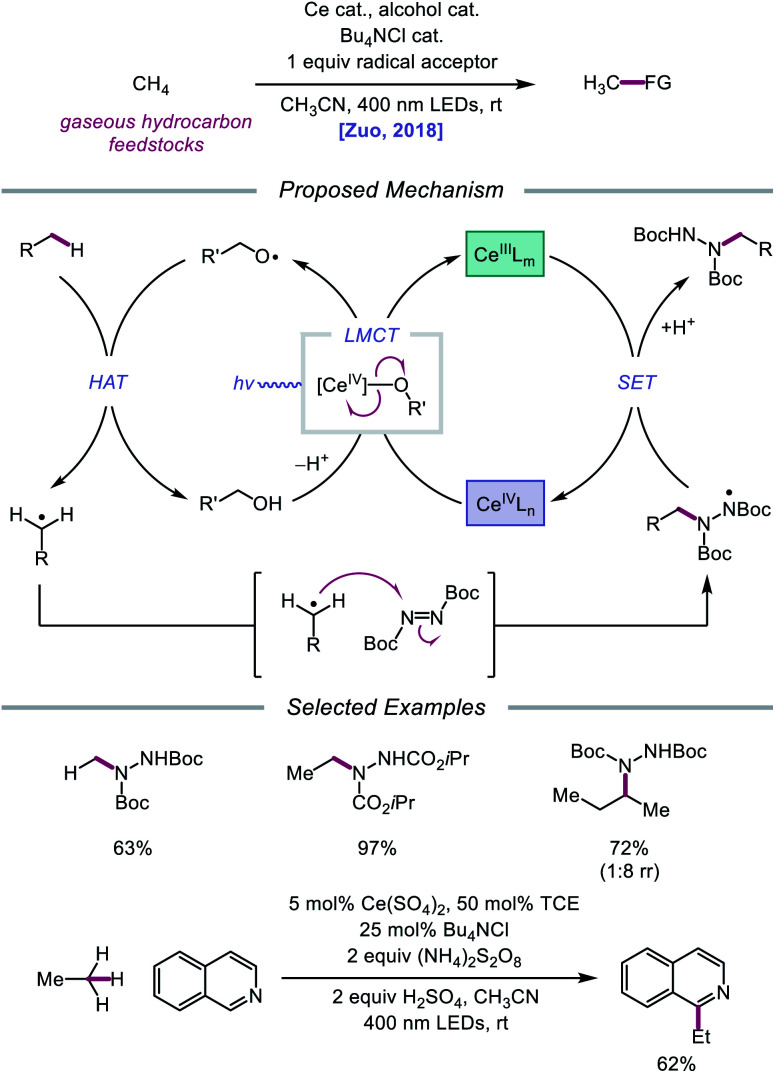
Cerium-catalyzed C–H functionalization of gaseous hydrocarbon feedstocks using alcohols as HAT catalysts.

Later that year, it was reported that alkoxy radicals generated from LMCT not only participate in intramolecular HAT but can also be used as intermolecular HAT catalysts for the C(sp^3^)–H bond functionalization of methane and other gaseous alkanes (Scheme 25).^75^ Recognizing that simple alcohols, such as methanol or 2,2,2-trichloroethanol (TCE), can serve as precursors to highly electrophilic alkoxy radicals, the authors propose that these catalysts can abstract an H atom from a methane C–H bond (C–H BDFE ≈ 97 kcal mol^–1^) to form an alkyl radical that proceeds to couple with an acceptor, such as DBAD.^12^,^13^ It is demonstrated that 0.5 mol% (Bu_4_N)_2_CeCl_6_ and 20 mol% TCE are effective at providing 63% yield for the amination of methane, while the use of 0.01 mol% CeCl_3_ and the same TCE catalyst leads to a remarkable yield of 97% aminated product with ethane. Propane and butane pose an interesting challenge with regards to selectivity between the slightly weaker methylene C–H bonds and the more numerous methyl C–H bonds. The authors observe that selectivity can be induced by modulating the electrophilicity of the alcohol HAT catalyst, with methanol generally favoring HAT from methylene C–H bonds. This mixed-phase gas/solution reaction has been adapted to a continuous flow platform, facilitating the scalable production of functionalized alkanes from gaseous hydrocarbon feedstocks.

**Scheme 26:**
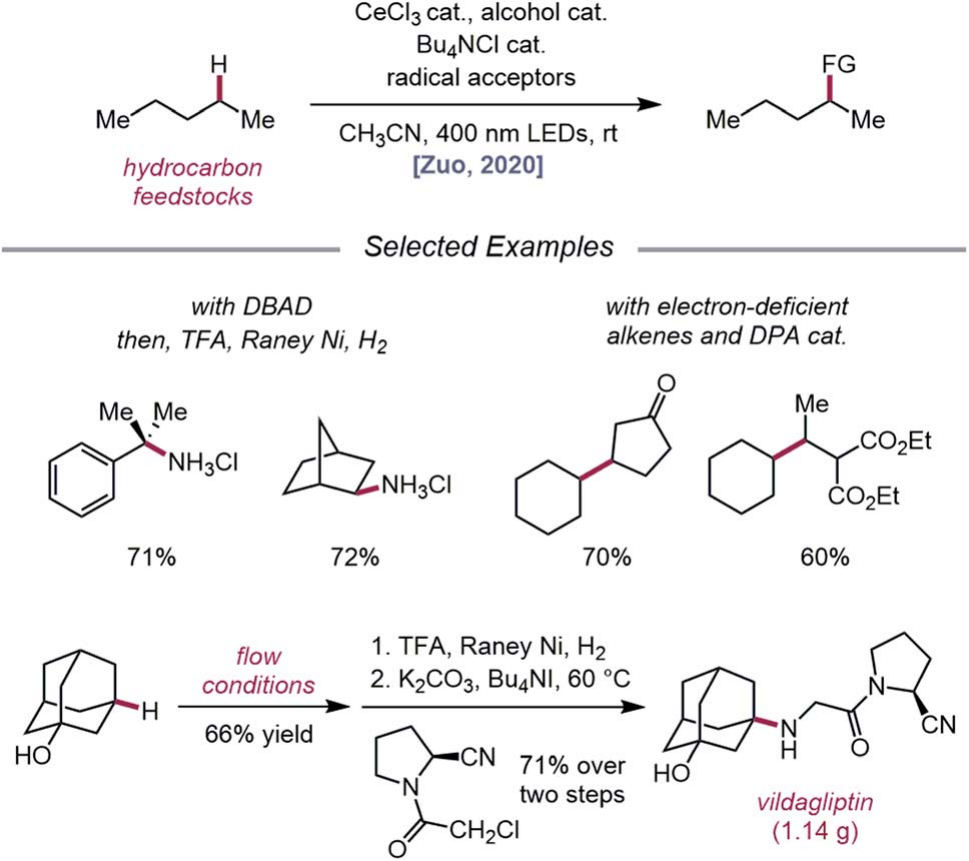
C–H functionalization of alkanes using alkoxy radical-mediated HAT.

Expanding upon this protocol for intermolecular HAT, Liu, Zuo, and coworkers in 2020 again utilized simple alcohols as tunable HAT catalysts for the C–H amination and alkylation of liquid hydrocarbons (Scheme 26).^76^ Extensive selectivity studies reveal that methanol-catalyzed conditions generally favor abstraction from weaker C–H bonds, while sterically bulky alcohols, such as *tert*-butanol or 1-adamantanol, promote abstraction from less sterically encumbered bonds. Electron-deficient alcohols, such as TCE or 2,2,2-trifluoroethanol, possess stronger O–H bonds and, thus, decrease selectivity for weaker C–H bonds when used as HAT catalysts. Guided by these principles, a variety of hydrocarbons, including cumene and norbornane, are successfully aminated with high selectivity for weak C–H bonds, while 1-adamantanol is converted to 3-amino-1-adamantanol on gram-scale *via* continuous flow and transformed into antidiabetic drug vildagliptin after two additional synthetic steps. Using DPA as a co-catalyst, C–H bond alkylation is also achieved with electron-deficient alkenes, from enones to substituted methylene-malonates. The LMCT event essential to the Zuo group's method for alkoxy radical generation was then investigated in detail using steady-state homolysis experiments and transient absorption spectroscopy. From this work, the quantum yield of cerium methoxide homolysis (38.8%) and the lifetime of the LMCT state (∼0.73 ps for cerium methoxide) are determined, collectively ruling out LMCT as the rate-determining step in this reaction.

## Proton-coupled electron transfer

As highlighted thus far, most modern methods for the activation of O–H bonds require the intermediacy of an alkoxyiodo or a metal–alkoxide species. In principle, direct HAT from the hydroxy group of alcohols would provide straightforward access to the desired alkoxy radical. However, the strength of such bonds (O–H BDFE ≈ 105 kcal mol^–1^) precludes the use of common hydrogen atom abstractors, which lack the necessary driving force to enable O–H activation.^10^,^77^ Moreover, unfavorable polar effects further limit the realization of HAT activation mechanisms—common H-atom acceptors are electrophilic in character and are kinetically slow to abstract from polarity-mismatched protic O–H bonds, instead preferentially activating weaker C–H bonds prevalent in organic molecules.^78^

**Scheme 27:**
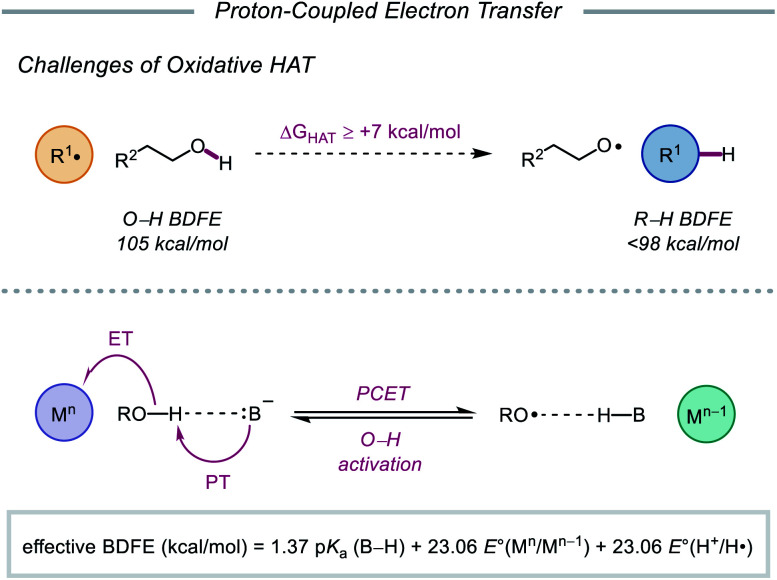
Challenges of conventional HAT, and multisite PCET mechanism for homolytic O–H activation.

In light of these limitations, the Knowles group sought to use proton-coupled electron transfer (PCET) as an alternative mechanism for direct O–H bond homolysis to provide catalytic access to alkoxy radicals from simple alcohol starting materials. Multisite PCET involves the concerted movement of a proton and an electron in a single elementary step to site-separated acceptors (Scheme 27).^79^ Unlike classical HAT, PCET allows access to a broader thermodynamic range through the use of tunable oxidant/base pairs that together serve as formal H-atom acceptors. Each catalyst pair can be described by an effective BDFE that represents the strength of the bond that can be broken in a thermoneutral reaction using this reagent system, calculated from the p*K*_a_ value of the conjugate acid (B–H) of the employed base, the reduction potential of the oxidant, and the reduction potential of H^+^ to H˙.^12^,79d,e,^80^ By modulating the p*K*_a_ of B–H and the reduction potential of the one-electron oxidant, effective BDFEs can be achieved that are thermodynamically competent to activate aliphatic O–H bonds, overcoming the energetic limitations of classical HAT reagents. Additionally, the selectivity of multi-site PCET events, which are dictated by hydrogen bonding between the Brønsted base and polar functional group, is expected to be orthogonal to HAT processes, as C–H bonds are poor hydrogen bond donors. Using a separated oxidant/base catalyst pair can enable polar O–H bonds to be homolyzed selectively. Along with these advantages, concerted PCET processes are often kinetically faster than the stepwise electron transfer or proton transfer steps. This is due to the fact that the kinetics of PCET are a function of the reaction's thermodynamic driving force.^81^ Since the products formed *via* a concerted pathway are lower in energy than the intermediates formed in the stepwise pathways, PCET activation of O–H bonds is expected to be more efficient than stepwise deprotonation/oxidation steps when relatively weak oxidants and bases are employed. In fact, PCET has long been implicated in the activation of phenols, particularly in biological systems,^82^ but prior to 2016, PCET activation of aliphatic O–H bonds had largely remained elusive.^83^

**Scheme 28:**
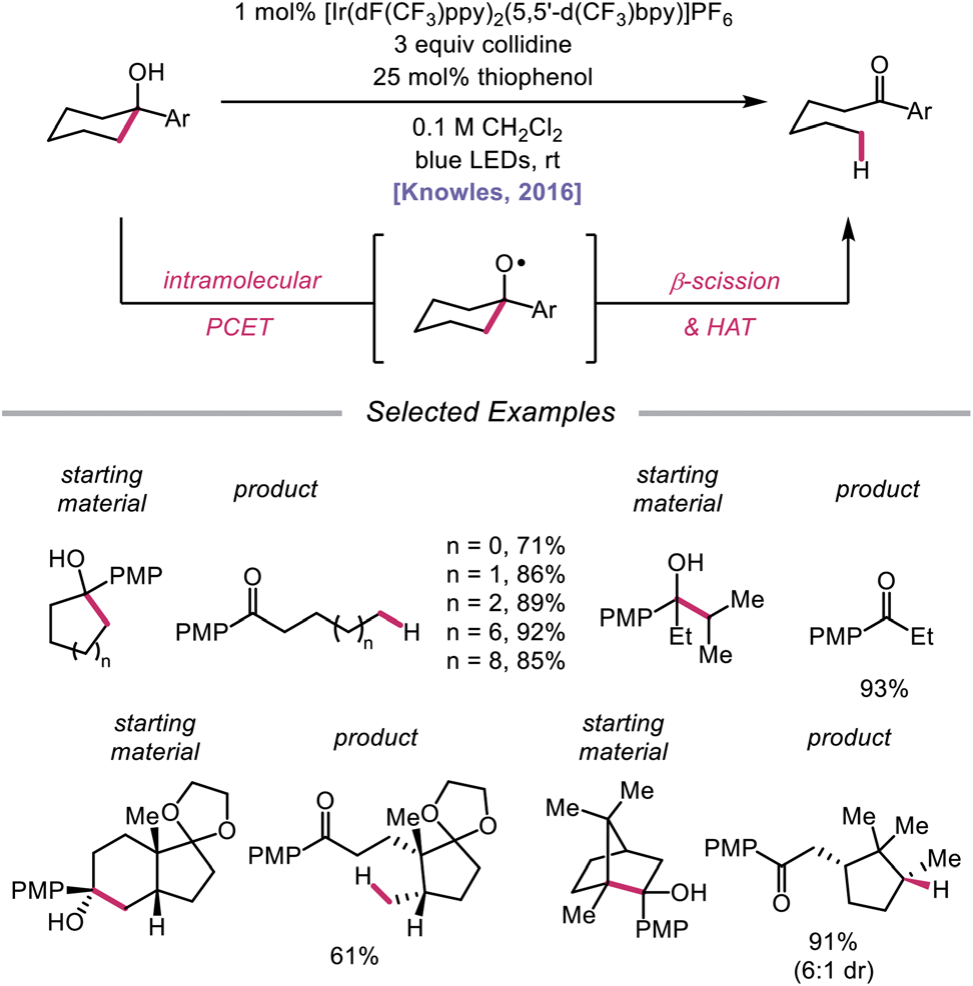
Ring-opening of cycloalkanols enabled by PCET activation of O–H bonds. PMP, *p*-methoxyphenyl.

In 2016, Knowles and coworkers leveraged this PCET framework for the direct catalytic activation of tertiary alcohol O–H bonds.^84^ Inspired by mechanistic studies from Baciocchi, Bietti, Lanzalunga, and Steenken,^85^ the authors achieved a redox-neutral isomerization of cyclic aryl alkanols to linear ketones through a C–C bond scission step mediated by an alkoxy radical intermediate (Scheme 28). This protocol for alkoxy radical generation relies on an intramolecular PCET event comprising deprotonation of a hydroxyl group by a Brønsted base in concert with one-electron reduction of a proximal arene radical cation. In particular, Baciocchi's studies suggest that this reaction design enables alcohol O–H bonds to be selectively deprotonated over C–H bonds at near diffusion-controlled rates,^85^ despite modest driving forces. Various tertiary cycloalkanols across a range of ring sizes can be accommodated, indicating that strained ring systems are not a requirement for β-scission under these conditions; linear alcohols that can extrude free alkyl radical fragments are also amenable to the reaction. Notably, this ring-opening strategy can also tolerate polycyclic structures without compromising proximal stereocenters. However, this catalytic system is limited to substrates decorated with a pendent electron-rich arene in order to facilitate PCET reactivity.

**Scheme 29:**
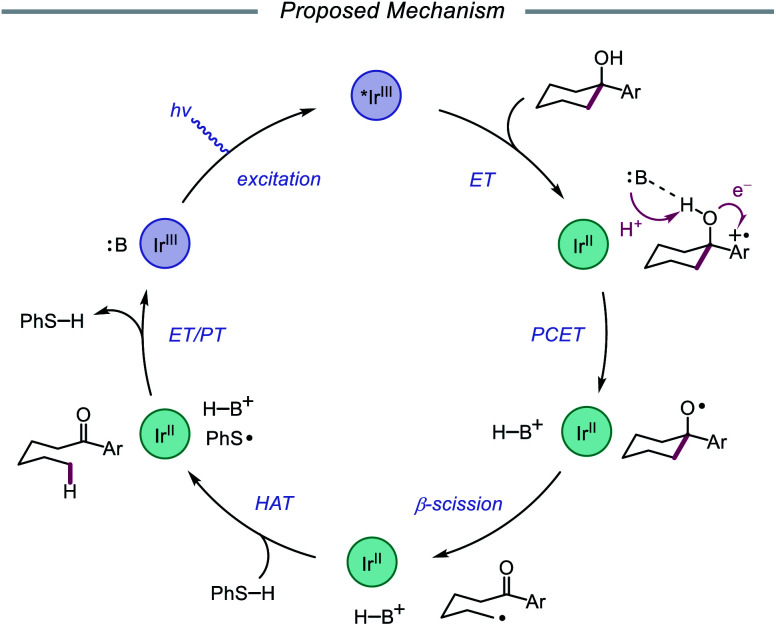
Proposed catalytic cycle for ring-opening isomerization of cyclic alcohols *via* PCET.

Results from mechanistic studies are consistent with the proposed catalytic cycle (Scheme 29), wherein an excited-state Ir(iii) photoredox catalyst oxidizes an electron-rich arene on the substrate to furnish a radical cation. An intramolecular PCET event then occurs, during which concerted deprotonation by collidine as the Brønsted base and oxidation by the proximal aryl radical cation as an internal oxidant affords the alkoxy radical. Subsequent β-fragmentation of the adjacent C–C bond results in ring-opening to generate an aryl ketone and a distal alkyl radical, which is then reduced by catalytic thiophenol. Finally, single electron reduction of the thiyl by the Ir(ii) species and protonation by collidinium turns over the catalytic cycle. Support for a PCET event in the formation of the alkoxy radical is drawn from an examination of the reaction efficiency for eight substrates bearing arenes with different oxidation potentials in the presence of four different Brønsted bases. These studies demonstrate that effective bond strength considerations can successfully predict the feasibility of alkoxy radical generation across the entire series. In 2018, the Yang group elaborated on this PCET system, demonstrating that the nascent alkyl radical formed after β-scission can be captured by radical acceptors as part of an allylation protocol or oxidized by O_2_ in air for subsequent formylation.^86^

**Scheme 30:**
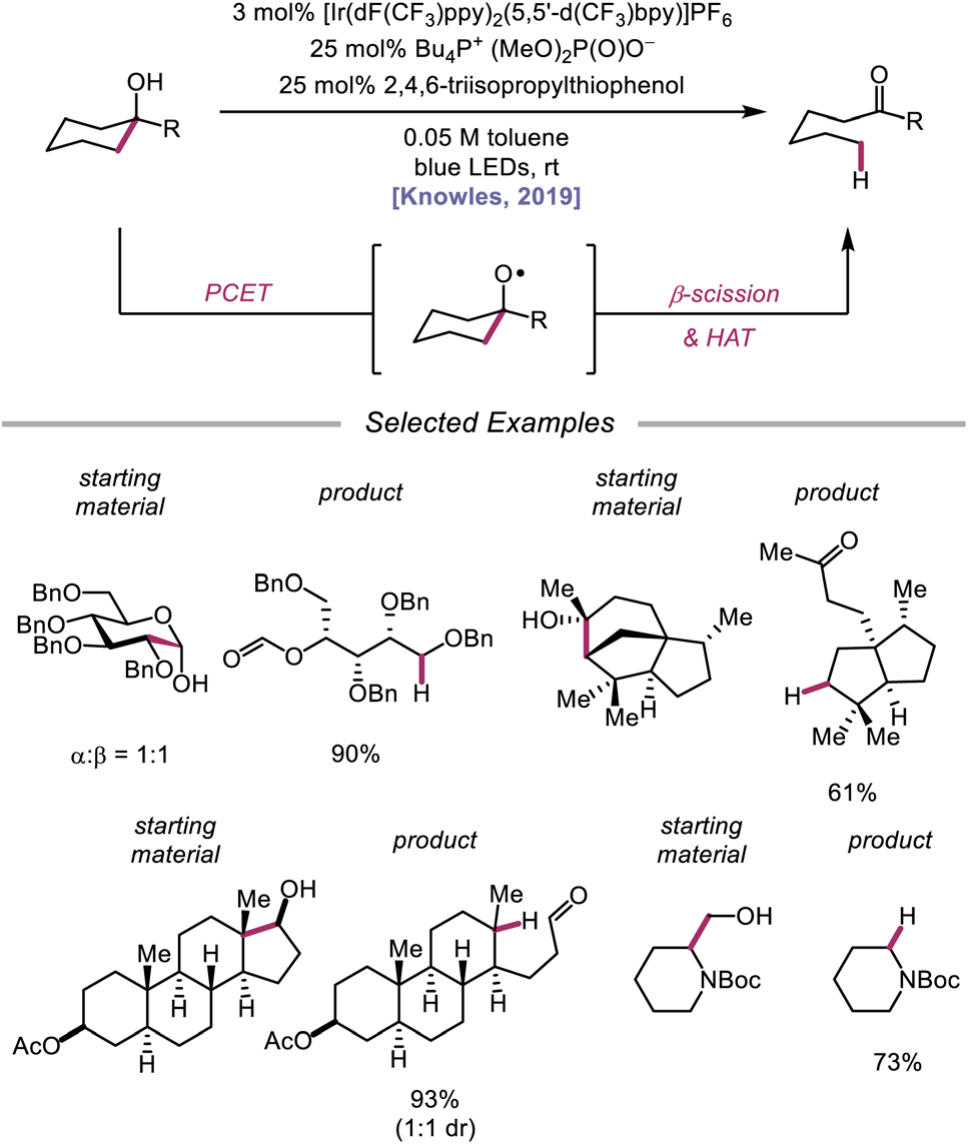
PCET activation of aliphatic alcohol O–H bonds and application to β-scission reactions.

**Scheme 31:**
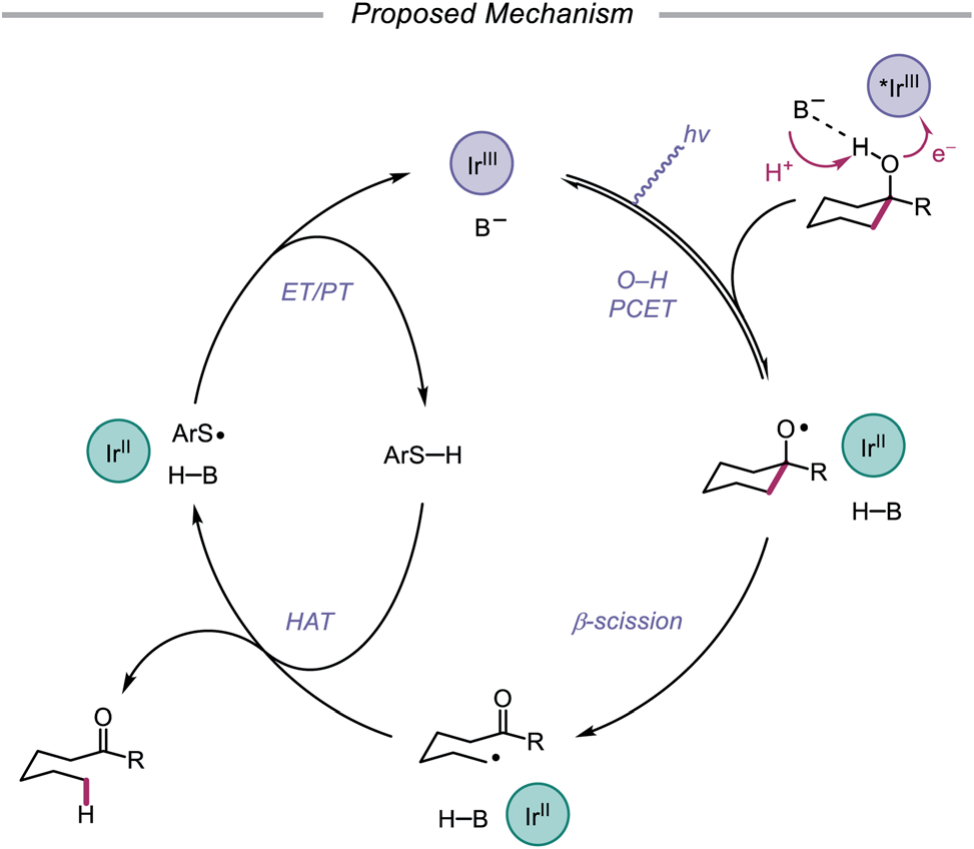
Proposed mechanism for C–C bond cleavage *via* direct PCET activation of alcohols.

In 2019, the Knowles group expanded upon the use of PCET as a catalytic platform for O–H activation by reporting a general procedure for the ring-opening isomerization of cyclic aliphatic alcohols (Scheme 30). Unlike the previous protocol, this strategy no longer requires that substrates bear an oxidizable arene.^87^ Instead, direct PCET activation of O–H bonds in primary, secondary, and tertiary aliphatic alcohols can be achieved with a key change—substituting the neutral collidine base for an anionic tetrabutylphosphonium dimethyl phosphate base. The authors hypothesize that the improved reaction efficiency may result from the enhanced ability of the anionic phosphate base to form a more favorable pre-PCET hydrogen bond complex with the alcohol substrate (Scheme 31). A wide range of alcohols and hemiacetals, including hexose and pentose derivatives, are viable substrates under these conditions, as well as more complex polycyclic scaffolds which can be isomerized with high regioselectivity. Interestingly, computational studies reveal that many of the cyclic alcohol starting materials are thermodynamically more stable than their ring-opened carbonyl products, even though the reaction is a redox-neutral isomerization that consumes no stoichiometric reagents other than photons. These observations demonstrate how excited-state electron transfer events can be employed to drive reactions against a thermodynamic gradient in a manner that cannot be accomplished using conventional ground-state catalysts. The PCET strategy described here for C–C bond scission was adapted independently by the groups of Zhang and Knowles for the depolymerization of lignin models and biomass-derived polymeric lignin.^88^,^89^

**Scheme 32:**
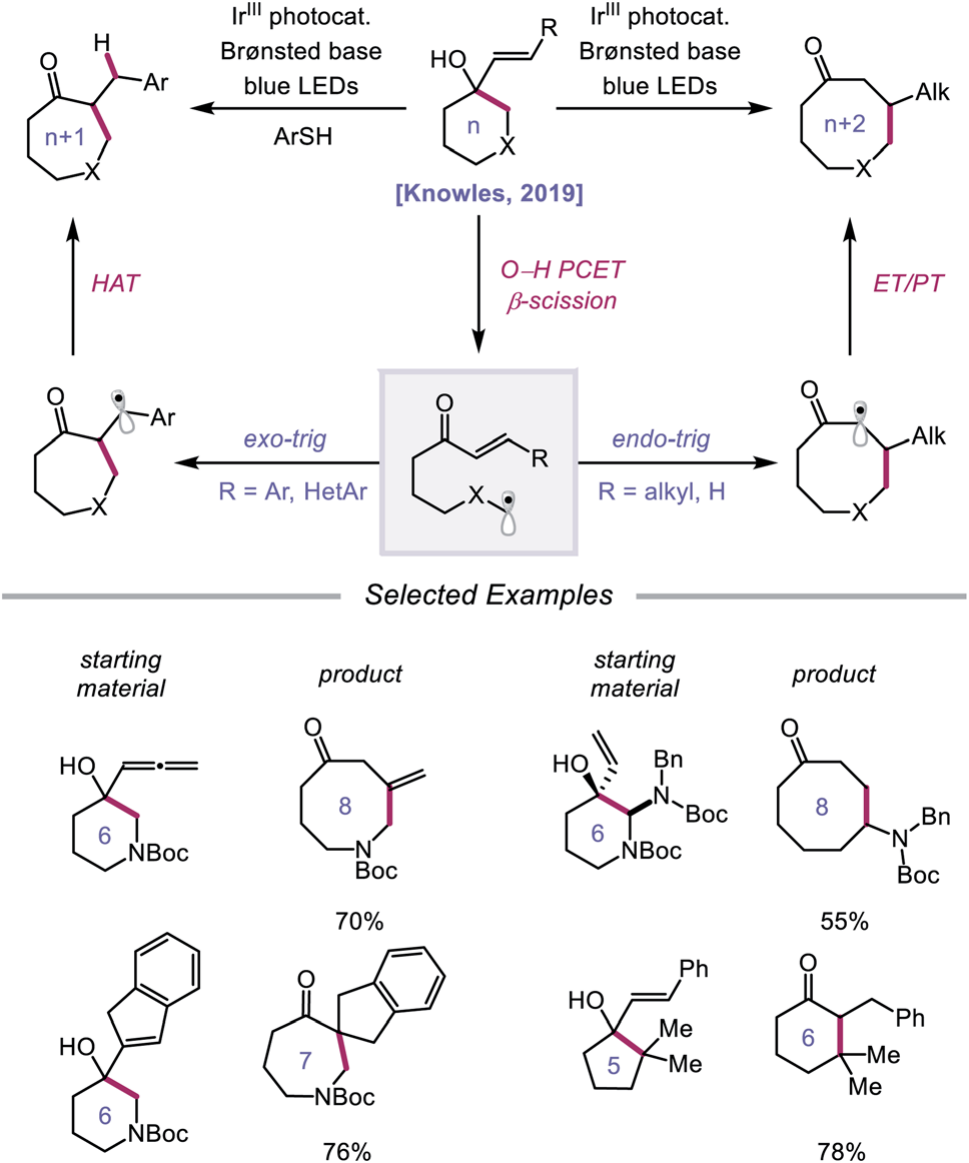
Catalytic ring expansion of cyclic allylic alcohols enabled by PCET.

Following their 2019 report, the Knowles lab developed a strategy for the catalytic ring expansion of cyclic alcohols to *n* + 1 or *n* + 2 expanded ketones by taking advantage of the propensity of alkoxy radicals to undergo C–C cleavage.^90^ By employing a similar series of elementary steps as in their prior work, O–H PCET is leveraged to generate an alkoxy radical intermediate from cyclic allylic alcohols (Scheme 32). After β-scission, an α,β-unsaturated ketone and tethered alkyl radical are formed, which recombine to give a new C–C bond as part of a ring-expanded ketone product. The regioselectivity of this enone addition step can be varied to allow access to ring systems homologated by two of the olefinic carbons (*n* + 2) or by only one carbon (*n* + 1). The selectivity of this C–C bond-forming step is controlled by the nature of the substituent on the exocyclic olefin acceptor, with alkyl substituents favoring *endo-trig* cyclization to generate an α-acyl radical and aryl substituents preferring *exo-trig* cyclization to furnish a stabilized benzylic radical. While the Ir(ii) state of the photocatalyst can adequately reduce the α-acyl radical and close the catalytic cycle, the authors note that in the latter *exo-trig* case, the potential of Ir(ii) is insufficient to reduce the benzylic radical. To overcome this complication, 2,4,6-triisopropylthiophenol is employed catalytically to mediate HAT reduction of the benzylic radical.

**Scheme 33:**
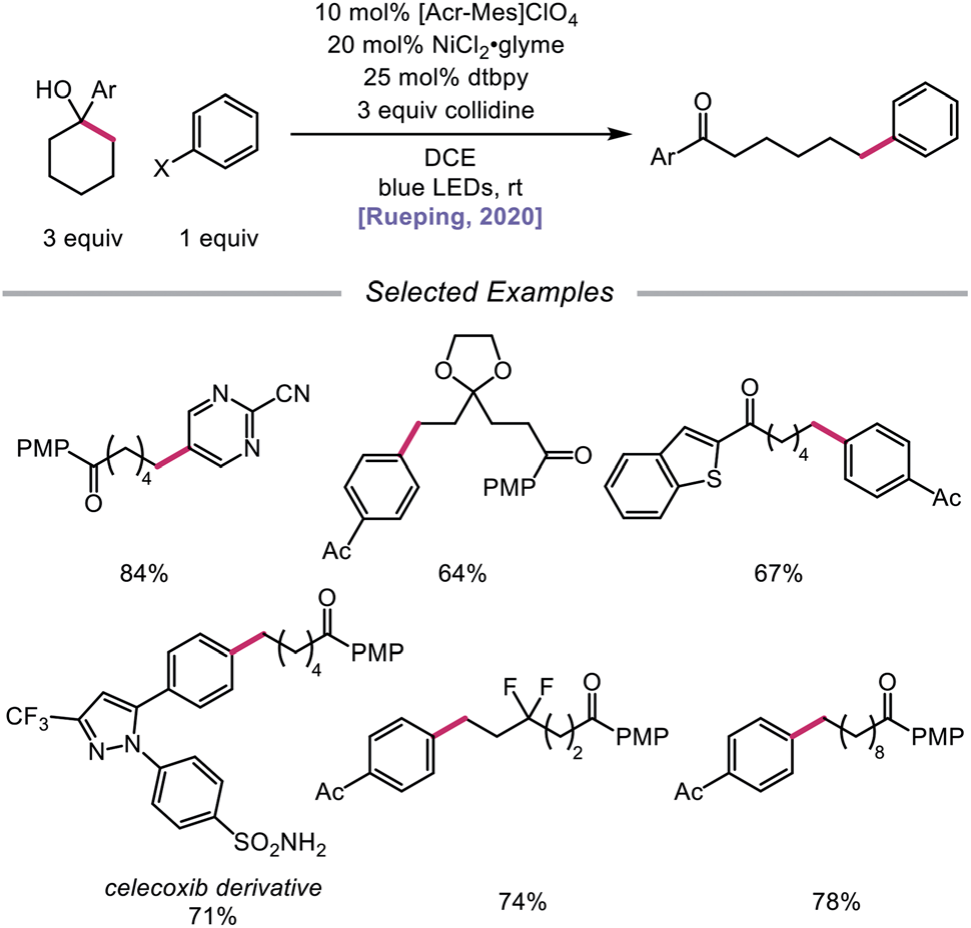
Nickel-catalyzed arylation of C–C bonds *via* PCET-promoted β-scission.

**Scheme 34:**
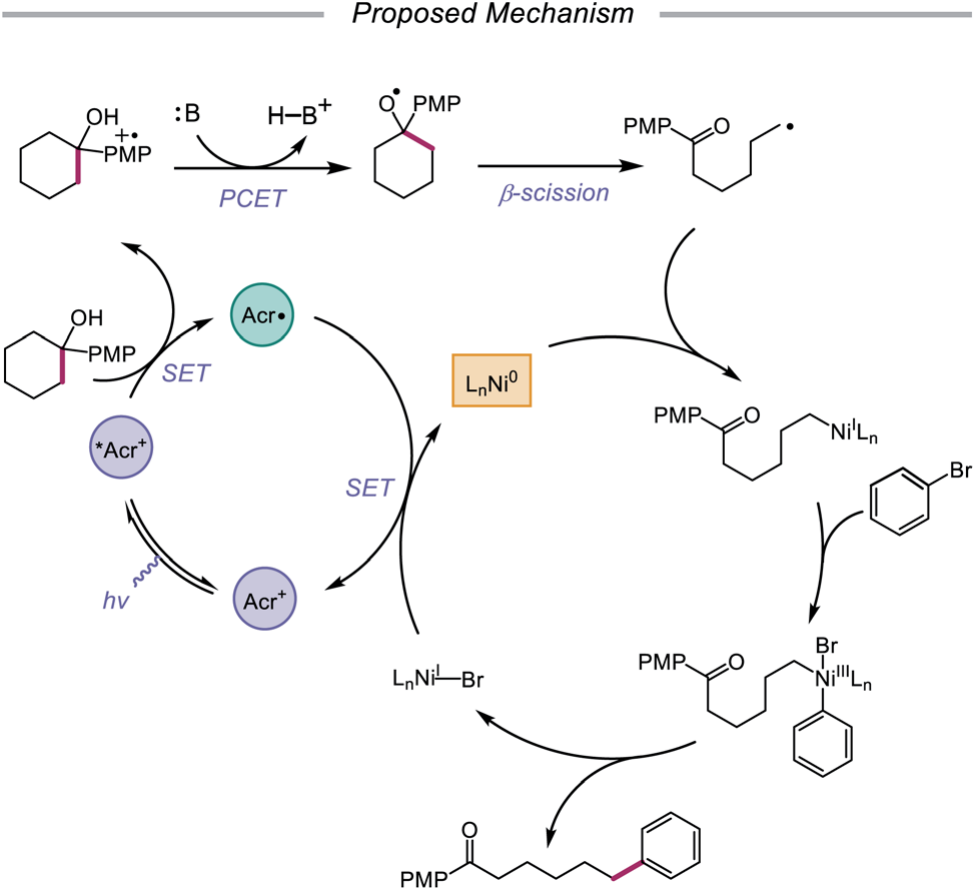
Postulated mechanism for site-specific arylation of ring-opened ketones enabled by PCET and nickel catalysis.

In 2020, the Rueping group reported a strategy using PCET-promoted β-scission to achieve site-specific nickel-catalyzed arylation of ketones (Scheme 33).^91^ The authors propose a mechanism wherein a highly oxidizing acridinium-derived organophotocatalyst and collidine base are used to activate the O–H bond in an arene-containing tertiary alcohol through a radical-cation redox relay PCET event (Scheme 34). Subsequent C–C bond cleavage provides a distal carbon-centered radical that can be intercepted by Ni(0) to give an alkylnickel(i) intermediate. Oxidative addition with an aryl halide affords a Ni(iii) species that can then undergo reductive elimination to furnish the cross-coupled product, creating a new C(sp^3^)–C(sp^2^) bond in the process. By merging PCET with nickel photoredox catalysis, Rueping and coworkers demonstrate that the site of arylation can be modulated by using carbocycles of various ring sizes, from 3-membered rings to 15-membered ring derivatives, regardless of the ring strain. Bridged-rings and tertiary linear alcohols are also competent radical precursors in this cross-coupling system. Following this report, the authors disclosed a related intramolecular PCET system that achieves trifluoromethylthiolation of ring-opened ketone products.^92^

**Scheme 35:**
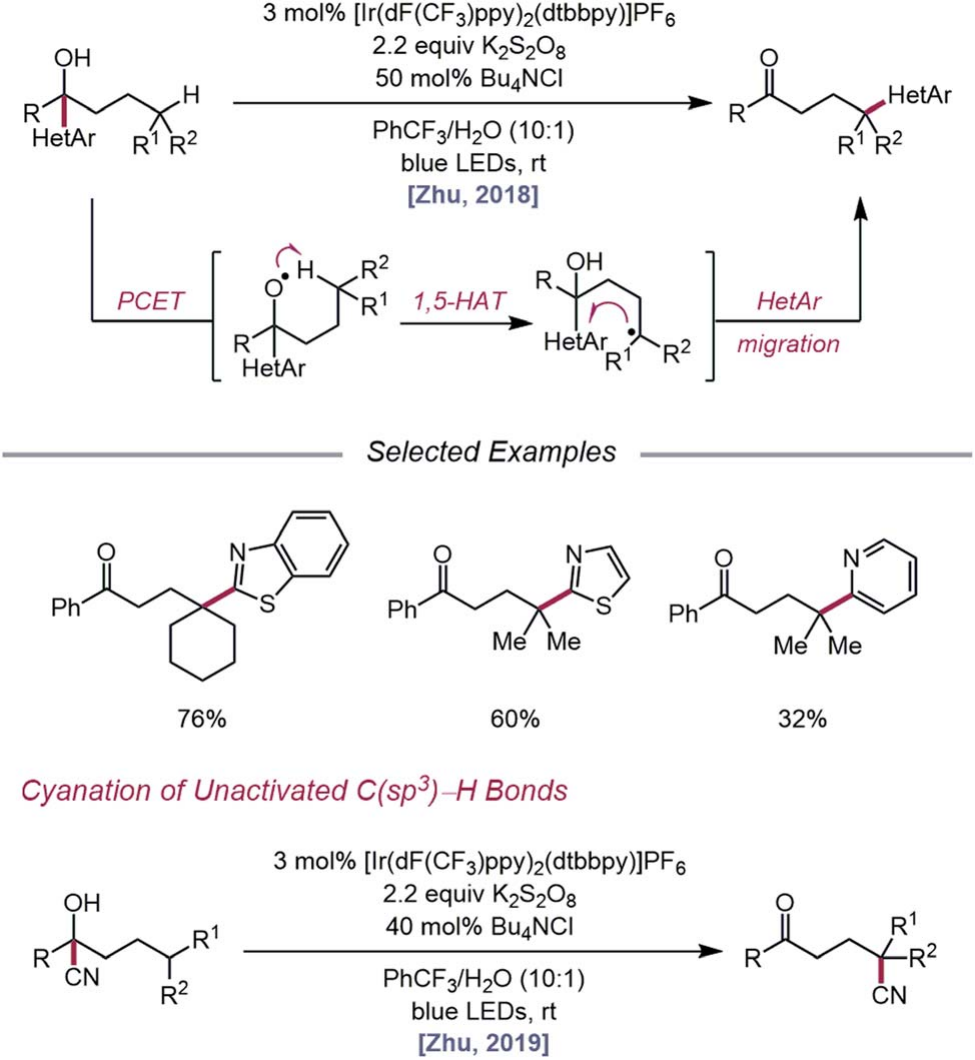
Alcohol-directed heteroarylation and cyanation of remote C(sp^3^)–H bonds.

As methods for the ring-opening and functionalization of cycloalkanols continued to be explored, Zhu and coworkers reported a strategy for the heteroarylation of remote C(sp^3^)–H bonds *via* an alkoxy radical intermediate.^93^ Starting from a heteroarene-containing tertiary alcohol in the presence of an Ir(iii) photocatalyst, K_2_S_2_O_8_, and Bu_4_NCl, a variety of isomeric heteroaryl ketones are formed, with γ-C–H bonds functionalized with migrated benzothiazolyl, thiazolyl, and pyridyl groups (Scheme 35). Stern–Volmer studies indicate that [Ir(dF(CF_3_)ppy)_2_(dtbbpy)]PF_6_ can be oxidatively quenched by K_2_S_2_O_8_. The inability of the resulting Ir(iv) complex (*E*_1/2_(IV/III) = 1.69 V *vs.* SCE in MeCN) to engage the alcohol substrate in direct electron transfer (*E*_*p*/2_ = 2.06 V *vs.* SCE in MeCN) leads the authors to postulate that a ground-state PCET process involving Ir(iv) as the electron acceptor may be operative. Once the alkoxy radical is formed, it triggers successive 1,5-HAT and intramolecular heteroaryl migration to furnish a ketone product. In a similar vein, the Zhu group disclosed a PCET process for cyanation of remote C–H bonds *via* intramolecular cyano group migration (Scheme 35).^94^ A PCET mechanism comprising Ir(iv) as the oxidant and sulfate as the base is invoked, although the authors note that direct HAT from the alcohol O–H bond by a sulfate radical anion cannot be ruled out.

**Scheme 36:**
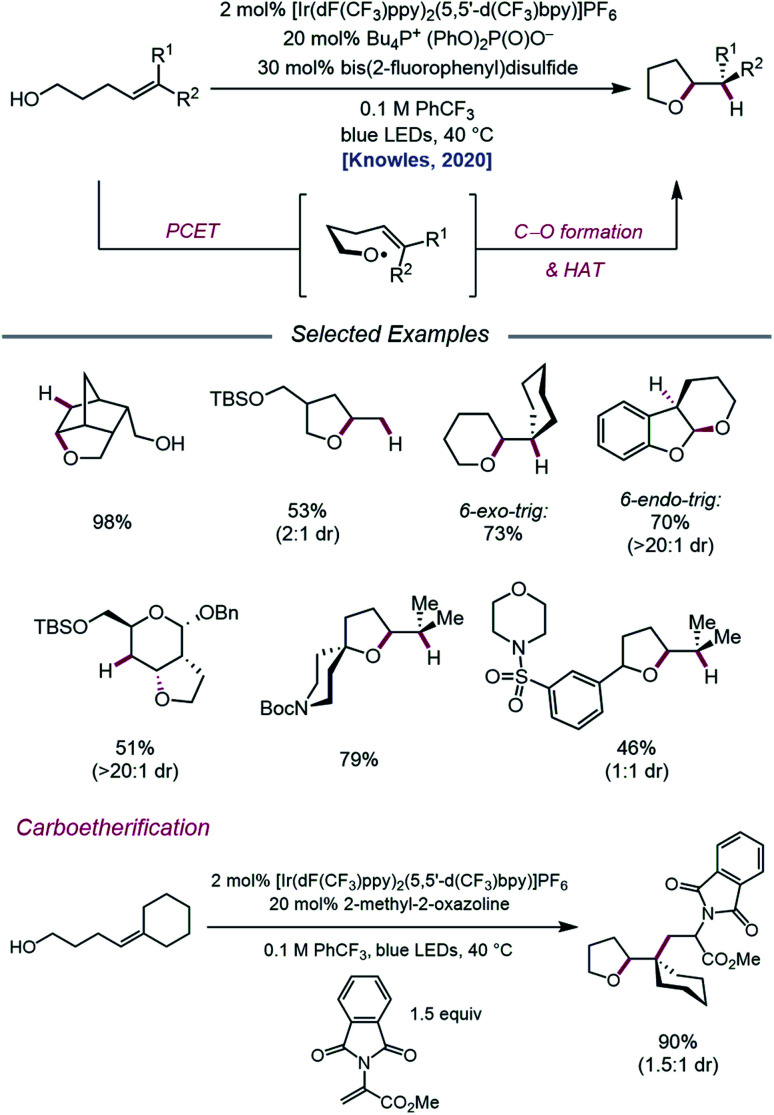
Catalytic hydroetherification and carboetherification of unactivated alkenes enabled by PCET.

In an effort to extend the synthetic applications of PCET activation of O–H bonds beyond β-fragmentation reactions, Knowles and coworkers reported in 2020 a catalytic system for the intramolecular hydroetherification and carboetherification of alkenols (Scheme 36).^95^ The authors propose a transformation in which the nascent electrophilic alkoxy radical generated from PCET adds into a pendent olefin, subsequently producing an adjacent C-centered radical that can be quenched with either an HAT co-catalyst to form tetrahydrofuran products or intercepted by an electron-deficient alkene to generate alkylated tetrahydrofurans. Trisubstituted, disubstituted, styrenyl, and monosubstituted alkenes are competent under the reaction conditions, and groups such as alkyl halides, heterocycles, sulfonamides, and thioethers are shown to be compatible. Notably, the versatility of this transformation gives rise to fused, bridged, and spirocyclic tetrahydrofuran scaffolds from simple alkenol starting materials. In addition to 5-*exo-trig* cyclizations, this method can also be utilized to achieve 6-*endo-trig* and 6-*exo-trig* ring closures with trisubstituted alkenes to provide tetrahydropyran products, with the latter outcompeting 1,5-HAT from allylic C–H bonds.

**Scheme 37:**
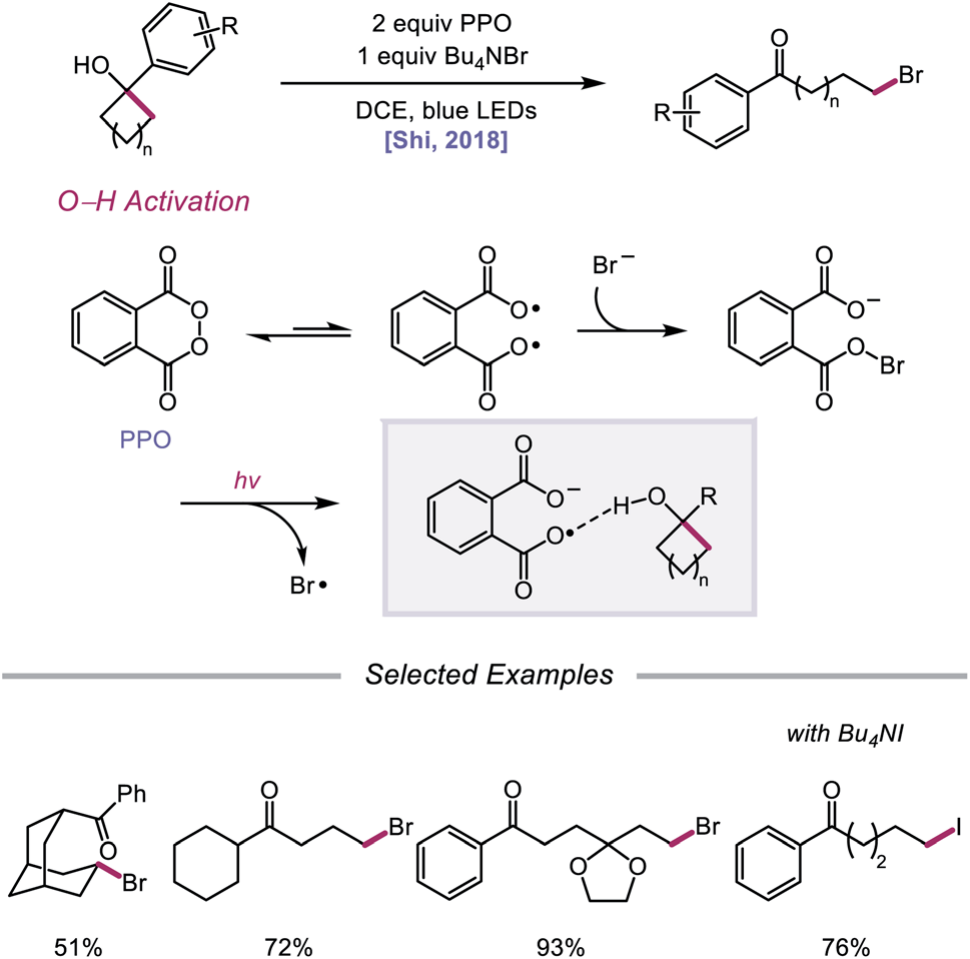
Ring-opening halogenation of cycloalkanols *via* HAT induced by Brønsted base-tethered acyloxy radicals.

Against this backdrop of new methods for PCET activation of alcohol O–H bonds, Shi and coworkers proposed in 2018 a related but alternative mechanism for alkoxy radical generation enabled by direct HAT.^96^ Using stoichiometric phthaloyl peroxide (PPO) and Bu_4_NBr under blue light irradiation, radical ring-opening and bromination of a variety of cycloalkanols are accomplished (Scheme 37). Based on kinetic isotope effect studies and DFT computations, the authors suggest that, rather than PCET, PPO facilitates the formation of an alkoxy radical intermediate through a direct HAT event aided by hydrogen-bonding interactions. PPO, in equilibrium with its diradical form, engages with a bromide anion to form a radical-anion intermediate, which has an enhanced ability to promote HAT from O–H bonds due to H-bonding between the carboxyl groups on PPO and the alcohol substrate. It is proposed that 5- to 8-membered cyclic alcohols undergo HAT to the PPO-derived radical-anion, producing an alkoxy radical that then undergoes β-scission to the ring-opened intermediate. Coupling with a bromine radical furnishes the distally brominated ketone product.

## Conclusions

Significant advances in catalytic alcohol O–H activation, largely enabled by the development of light-driven and transition metal-catalyzed mechanisms, have facilitated straightforward access to alkoxy radicals from free alcohols and have provided a solution to a long-standing challenge in radical chemistry. By precluding the need for prefunctionalized substrates and stoichiometric reagents, this diverse repertoire of methods has been utilized for the development of myriad transformations mediated by alkoxy radicals, including C–C bond cleavage, remote C–H bond functionalization, and C–O bond formation. While these strategies for alkoxy radical generation have expanded the scope of new transformations and inspired novel disconnections, several challenges remain. The next steps in the advancement of these catalytic systems are to control the fate of the alkoxy radical by modulating reactivity toward HAT, β-scission, or alkene addition *via* catalyst control, expanding the scope of intermolecular reactivity, and developing technologies for stereocontrol in alkoxy radical-mediated transformations. We anticipate that progress in these areas can be achieved as we continue to see innovations in alkoxy radical chemistry in the years to come.

## Conflicts of interest

There are no conflicts to declare.

## References

[cit1] Wieland H. (1911). Ber. Dtsch. Chem. Ges..

[cit2] Gray P., Williams A. (1959). Chem. Rev..

[cit3] Rueda-Becerril M., Leung J. C. T., Dunbar C. R., Sammis G. M. (2011). J. Org. Chem..

[cit4] Majetich G., Wheless K. (1995). Tetrahedron.

[cit5] Čeković ž. (2003). Tetrahedron.

[cit6] Suárez E. and Rodriguez M. S., in Radicals in Organic Synthesis, ed. P. Renaud and M. P. Sibi, Wiley-VCH Verlag GmbH, Weinheim, Germany, 2001, pp. 440–454.

[cit7] Murakami M., Ishida N. (2017). Chem. Lett..

[cit8] Hartung J. (2001). Eur. J. Org. Chem..

[cit9] Hartung J., in Radicals in Organic Synthesis, ed. P. Renaud and M. P. Sibi, Wiley-VCH Verlag GmbH, Weinheim, Germany, 2001, pp. 427–439.

[cit10] Feray L., Kuznetsov N. and Renaud P., in Radicals in Organic Synthesis, ed. P. Renaud and M. P. Sibi, Wiley-VCH Verlag GmbH, Weinheim, Germany, 2001, pp. 246–278.

[cit11] Salamone M., Bietti M. (2015). Acc. Chem. Res..

[cit12] Warren J. J., Tronic T. A., Mayer J. M. (2010). Chem. Rev..

[cit13] Blanksby S. J., Ellison G. B. (2003). Acc. Chem. Res..

[cit14] Barton D. H. R., Beaton J. M., Geller L. E., Pechet M. M. (1960). J. Am. Chem. Soc..

[cit15] Kochi J. K. (1962). J. Am. Chem. Soc..

[cit16] Morcillo S. P. (2019). Angew. Chem., Int. Ed..

[cit17] Yu X. Y., Chen J. R., Xiao W. J. (2020). Chem. Rev..

[cit18] Zhu H., Leung J. C. T., Sammis G. M. (2015). J. Org. Chem..

[cit19] Horner J. H., Choi S. Y., Newcomb M. (2000). Org. Lett..

[cit20] de Armas P., Francisco C. G., Suárez E. (1993). J. Am. Chem. Soc..

[cit21] Surzur J. M., Bertrand M. P., Nouguier R. (1969). Tetrahedron Lett..

[cit22] Čeković Z., Cvetković M. (1982). Tetrahedron Lett..

[cit23] Walling C., Padwa A. (1961). J. Am. Chem. Soc..

[cit24] Beckwith A. L. J., Hay B. P., Williams G. M. (1989). J. Chem. Soc., Chem. Commun..

[cit25] Beckwith A. L. J., Hay B. P. (1988). J. Am. Chem. Soc..

[cit26] Kim S., Lee T. A., Song Y. (1998). Synlett.

[cit27] Zhang J., Li Y., Zhang F., Hu C., Chen Y. (2016). Angew. Chem., Int. Ed..

[cit28] Wang C., Harms K., Meggers E. (2016). Angew. Chem., Int. Ed..

[cit29] Barthelemy A. L., Tuccio B., Magnier E., Dagousset G. (2018). Angew. Chem., Int. Ed..

[cit30] Kim I., Park B., Kang G., Kim J., Jung H., Lee H., Baik M.-H., Hong S. (2018). Angew. Chem., Int. Ed..

[cit31] Bao X., Wang Q., Zhu J. (2019). Angew. Chem., Int. Ed..

[cit32] Capaldo L., Ravelli D. (2019). Chem. Commun..

[cit33] Herron A. N., Liu D., Xia G., Yu J.-Q. (2020). J. Am. Chem. Soc..

[cit34] Togo H., Katohgi M. (2001). Synlett.

[cit35] Concepción J. I., Francisco C. G., Hernández R., Salazar J. A., Suárez E. (1984). Tetrahedron Lett..

[cit36] Jia K., Zhang F., Huang H., Chen Y. (2016). J. Am. Chem. Soc..

[cit37] Jia K., Pan Y., Chen Y. (2017). Angew. Chem., Int. Ed..

[cit38] Norrish R. G. W., Bamford C. H. (1936). Nature.

[cit39] Souillart L., Cramer N. (2015). Chem. Rev..

[cit40] Jia K., Li J., Chen Y. (2018). Chem. - Eur. J..

[cit41] Wang D., Mao J., Zhu C. (2018). Chem. Sci..

[cit42] Wu X., Zhang H., Tang N., Wu Z., Wang D., Ji M., Xu Y., Wang M., Zhu C. (2018). Nat. Commun..

[cit43] Li G.-X., Hu X., He G., Chen G. (2019). Chem. Sci..

[cit44] Hu X., Li G.-X., He G., Chen G. (2019). Org. Chem. Front..

[cit45] Schaafsma S. E., Steinberg H., de Boer T. J. (1966). Recl. Trav. Chim. Pays-Bas.

[cit46] Meyer K., Roček J. (1972). J. Am. Chem. Soc..

[cit47] Iwasawa N., Hayakawa S., Isobe K., Narasaka K. (1991). Chem. Lett..

[cit48] Clerici A., Minisci F., Ogawa K., Surzur J. M. (1978). Tetrahedron Lett..

[cit49] Chiba S., Cao Z., El Bialy S. A. A., Narasaka K. (2006). Chem. Lett..

[cit50] Zhao H., Fan X., Yu J., Zhu C. (2015). J. Am. Chem. Soc..

[cit51] For recent work on silver-catalyzed β-scission and radical fluorination, see: Zhou X. Ding H. Chen P. Liu L. Sun Q. Wang X. Wang P. Lv Z. Li M., Angew. Chem., Int. Ed., 2020, 59 , 4138 –4144 . 10.1002/anie.201914557 31850616

[cit52] Ren S., Feng C., Loh T. P. (2015). Org. Biomol. Chem..

[cit53] Ishida N., Okumura S., Nakanishi Y., Murakami M. (2015). Chem. Lett..

[cit54] (b) Raclea R.-C. Natho P. Allen L. A. T. White A. J. P. Parsons P. J., J. Org. Chem., 2020, 85 , 9375 –9385 , ; see also: . 32543189 10.1021/acs.joc.0c00986

[cit55] Ren R., Zhao H., Huan L., Zhu C. (2015). Angew. Chem., Int. Ed..

[cit56] Yu J., Zhao H., Liang S., Bao X., Zhu C. (2015). Org. Biomol. Chem..

[cit57] Ren R., Wu Z., Xu Y., Zhu C. (2016). Angew. Chem., Int. Ed..

[cit58] Huan L., Zhu C. (2016). Org. Chem. Front..

[cit59] Wang D., Ren R., Zhu C. (2016). J. Org. Chem..

[cit60] Ren R., Wu Z., Zhu C. (2016). Chem. Commun..

[cit61] Wang M., Wu Z., Zhu C. (2017). Org. Chem. Front..

[cit62] Zhu Y., Huang K., Pan J., Qiu X., Luo X., Qin Q., Wei J., Wen X., Zhang L., Jiao N. (2018). Nat. Commun..

[cit63] Zhu Y., Zhang Z., Jin R., Liu J., Liu G., Han B., Jiao N. (2020). Angew. Chem., Int. Ed..

[cit64] Ren H., Song J.-R., Li Z.-Y., Pan W.-D. (2019). Org.Org.
Lett.Lett..

[cit65] (a) Chen D., Berhane I. A. and Chemler S. R., Org. Lett., 2020, 10.1021/acs.orglett.0c01691. PMC7541751 32496794

[cit66] Prier C. K., Rankic D. A., MacMillan D. W. C. (2013). Chem. Rev..

[cit67] Balzani V., Ceroni P. and Juris A., Photochemistry and Photophysics: Concepts, Research, Applications, Wiley-VCH, Weinheim, Germany, 2014.

[cit68] Yin H., Carroll P. J., Anna J. M., Schelter E. J. (2015). J. Am. Chem. Soc..

[cit69] Guo J.-J., Hu A., Chen Y., Sun J., Tang H., Zuo Z. (2016). Angew. Chem., Int. Ed..

[cit70] Young L. B., Trahanovsky W. S. (1969). J. Am. Chem. Soc..

[cit71] Chen Y., Du J., Zuo Z. (2020). Chem.

[cit72] Hu A., Chen Y., Guo J. J., Yu N., An Q., Zuo Z. (2018). J. Am. Chem. Soc..

[cit73] Zhang K., Chang L., An Q., Wang X., Zuo Z. (2019). J. Am. Chem. Soc..

[cit74] Hu A., Guo J.-J., Pan H., Tang H., Gao Z., Zuo Z. (2018). J. Am. Chem. Soc..

[cit75] Hu A., Guo J.-J., Pan H., Zuo Z. (2018). Science.

[cit76] An Q., Wang Z., Chen Y., Wang X., Zhang K., Pan H., Liu W., Zuo Z. (2020). J. Am. Chem. Soc..

[cit77] Green M. T., Dawson J. H., Gray H. B. (2004). Science.

[cit78] Roberts B. P. (1999). Chem. Soc. Rev..

[cit79] Weinberg D. R., Gagliardi C. J., Hull J. F., Murphy C. F., Kent C. A., Westlake B. C., Paul A., Ess D. H., McCafferty D. G., Meyer T. J. (2012). Chem. Rev..

[cit80] Bordwell F. G., Cheng J.-P., Harrelson J. A. (1988). J. Am. Chem. Soc..

[cit81] Mayer J. M. (2011). Acc. Chem. Res..

[cit82] Reece S. Y., Nocera D. G. (2009). Annu. Rev. Biochem..

[cit83] For an example of PCET activation of aliphatic O–H bonds, see: Wang D. Farquhar E. R. Stubna A. Münck E. Que L., Nat. Chem., 2009, 1 , 145 –150 . 19885382 10.1038/nchem.162 PMC2744316

[cit84] Yayla H. G., Wang H., Tarantino K. T., Orbe H. S., Knowles R. R. (2016). J. Am. Chem. Soc..

[cit85] Baciocchi E., Bietti M., Steenken S. (1997). J. Am. Chem. Soc..

[cit86] Wang J., Huang B., Shi C., Yang C., Xia W. (2018). J. Org. Chem..

[cit87] Ota E., Wang H., Frye N. L., Knowles R. R. (2019). J. Am. Chem. Soc..

[cit88] Wang Y., Liu Y., He J., Zhang Y. (2019). Sci. Bull..

[cit89] Nguyen S. T., Murray P. R. D., Knowles R. R. (2020). ACS Catal..

[cit90] Zhao K., Yamashita K., Carpenter J. E., Sherwood T. C., Ewing W. R., Cheng P. T. W., Knowles R. R. (2019). J. Am. Chem. Soc..

[cit91] Huang L., Ji T., Rueping M. (2020). J. Am. Chem. Soc..

[cit92] Ji T., Chen X. Y., Huang L., Rueping M. (2020). Org. Lett..

[cit93] Wu X., Wang M., Huan L., Wang D., Wang J., Zhu C. (2018). Angew. Chem., Int. Ed..

[cit94] Wang M., Huan L., Zhu C. (2019). Org. Lett..

[cit95] Tsui E., Metrano A. J., Tsuchiya Y., Knowles R. R. (2020). Angew. Chem., Int. Ed..

[cit96] Zhao R., Yao Y., Zhu D., Chang D., Liu Y., Shi L. (2018). Org. Lett..

